# The MexTAg collaborative cross: host genetics affects asbestos related disease latency, but has little influence once tumours develop

**DOI:** 10.3389/ftox.2024.1373003

**Published:** 2024-04-17

**Authors:** Scott A. Fisher, Kimberley Patrick, Tracy Hoang, Elly Marcq, Kiarash Behrouzfar, Sylvia Young, Timothy J. Miller, Bruce W. S. Robinson, Raphael Bueno, Anna K. Nowak, W. Joost Lesterhuis, Grant Morahan, Richard A. Lake

**Affiliations:** ^1^ National Centre for Asbestos Related Diseases (NCARD), Perth, WA, Australia; ^2^ School of Biomedical Sciences, The University of Western Australia, Perth, WA, Australia; ^3^ Institute for Respiratory Health, University of Western Australia, Perth, WA, Australia; ^4^ Center for Oncological Research (CORE), University of Antwerp, Antwerp, Belgium; ^5^ Lab of Dendritic Cell Biology and Cancer Immunotherapy, VIB Center for Inflammation Research, Brussels, Belgium; ^6^ Brussels Center for Immunology, Vrije Universiteit Brussel, Brussels, Belgium; ^7^ Centre for Diabetes Research, Harry Perkins Institute of Medical Research, Perth, WA, Australia; ^8^ Medical School, The University of Western Australia, Perth, WA, Australia; ^9^ Division of Thoracic Surgery, The Lung Center and the International Mesothelioma Program, Brigham and Women’s Hospital and Harvard Medical School, Boston, MA, United States

**Keywords:** mesothelioma, collaborative cross, MexTAg, asbestos related disease, host genetics, mouse model, gene discovery

## Abstract

**Objectives:** This study combines two innovative mouse models in a major gene discovery project to assess the influence of host genetics on asbestos related disease (ARD). Conventional genetics studies provided evidence that some susceptibility to mesothelioma is genetic. However, the identification of host modifier genes, the roles they may play, and whether they contribute to disease susceptibility remain unknown. Here we report a study designed to rapidly identify genes associated with mesothelioma susceptibility by combining the Collaborative Cross (CC) resource with the well-characterised MexTAg mesothelioma mouse model.

**Methods:** The CC is a powerful mouse resource that harnesses over 90% of common genetic variation in the mouse species, allowing rapid identification of genes mediating complex traits. MexTAg mice rapidly, uniformly, and predictably develop mesothelioma, but only after asbestos exposure. To assess the influence of host genetics on ARD, we crossed 72 genetically distinct CC mouse strains with MexTAg mice and exposed the resulting CC-MexTAg (CCMT) progeny to asbestos and monitored them for traits including overall survival, the time to ARD onset (latency), the time between ARD onset and euthanasia (disease progression) and ascites volume. We identified phenotype-specific modifier genes associated with these traits and we validated the role of human orthologues in asbestos-induced carcinogenesis using human mesothelioma datasets.

**Results:** We generated 72 genetically distinct CCMT strains and exposed their progeny (2,562 in total) to asbestos. Reflecting the genetic diversity of the CC, there was considerable variation in overall survival and disease latency. Surprisingly, however, there was no variation in disease progression, demonstrating that host genetic factors do have a significant influence during disease latency but have a limited role once disease is established. Quantitative trait loci (QTL) affecting ARD survival/latency were identified on chromosomes 6, 12 and X. Of the 97-protein coding candidate modifier genes that spanned these QTL, eight genes (*CPED1, ORS1, NDUFA1, HS1BP3, IL13RA1, LSM8, TES* and *TSPAN12*) were found to significantly affect outcome in both CCMT and human mesothelioma datasets.

**Conclusion:** Host genetic factors affect susceptibility to development of asbestos associated disease. However, following mesothelioma establishment, genetic variation in molecular or immunological mechanisms did not affect disease progression. Identification of multiple candidate modifier genes and their human homologues with known associations in other advanced stage or metastatic cancers highlights the complexity of ARD and may provide a pathway to identify novel therapeutic targets.

## 1 Introduction

Mesothelioma is an aggressive cancer with poor prognosis, etiologically linked to asbestos exposure (Wagner et al., 1960; Robinson and Lake, 2005; Ramada Rodilla et al., 2022). Mesothelioma development is characterised by long latency periods with disease often taking 20–40 years to manifest after initial exposure. Despite many countries having banned the mining and use of asbestos-containing products in the early to mid-2000s, a significant exposure risk remains due to the presence of asbestos persisting in the open and built environment. This risk is further compounded by the continued mining and use of asbestos products in many populous, industrializing nations. Consequently, asbestos exposure and the subsequent risk of asbestos related disease remains a significant global health burden (Odgerel et al., 2017; GBDCoD, 2018).

Mesothelioma development after asbestos exposure is highly variable: some people do not develop disease despite high level exposure for many years, while others develop disease with no known history of exposure. At least part of the difference in susceptibility to mesothelioma is genetic: familial development of mesothelioma has been identified in patients with BAP1 syndrome (Carbone et al., 2013), as well as germline mutations in other DNA repair genes including *PALB2, BRCA1, FANCI, ATM, SLX4, BRCA2, FANCC, FANCF, PMS1* and *XPC CHEK2* (Betti et al., 2017), with known germline mutations identified in approximately 12% of patients (Panou et al., 2018). While much is known about somatic mutations associated with mesothelioma, the role of host genetics in disease development is less well understood.

Identifying genetic risk factors for mesothelioma development using conventional analyses has proven difficult. We previously performed a GWAS in which 2,508,203 single nucleotide polymorphisms (SNPs) from 428 confirmed mesothelioma cases from the asbestos mining town Wittenoom and 1,269 controls from an ongoing general population cohort study of residents from the town of Busselton, Western Australia (James et al., 2010) were compared (Cadby et al., 2013). Despite this study suggesting a contribution of genetic variation to mesothelioma risk in three loci (*SDK1, CRTAM and RAS-GRF2*), these data were not replicated in an independent case -control cohort (Matullo et al., 2013). Similar outcomes have been observed for other GWAS that have attempted to identify mesothelioma susceptibility genes. While each study identified gene variants with significant associations with asbestos exposure, GWAS have consistently failed to identify common genetic risk factors; suggesting that the variants identified in these studies are likely cohort-specific, with an overall minor impact on disease risk (Roe et al., 2009; Roe et al., 2010; Tunesi et al., 2015; Borczuk et al., 2016). Furthermore, it remains unknown where the identified genes act in the disease pathway, or how they contribute to disease pathogenesis. Taken together, these studies highlight limitations in the use of conventional genetic studies to identify host-gene interactions that affect rare cancers like mesothelioma.

To address these challenges, we developed a unique mouse model, the MexTAg Collaborative Cross (CCMT) (Behrouzfar et al., 2021). We combined the genetically diverse Collaborative Cross (CC), with the well-characterised MexTAg mesothelioma mouse model (Robinson et al., 2006; Robinson et al., 2011). The CC is a powerful mouse genetic resource specifically developed for rapid identification of genes associated with complex polygenic traits (Churchill et al., 2004; Chesler et al., 2008; Iraqi et al., 2008; Morahan et al., 2008; Collaborative Cross Consortium, 2012; Welsh et al., 2012). The CC consists of a collection of hundreds of recombinant inbred mouse strains developed from eight founder strains selected to maximize genetic diversity (Morahan et al., 2008). Each strain has a mosaic of genetic polymorphisms inherited from the founders and has the advantage over conventional genetic studies in that avoids the need for genotyping. A further advantage of using the CC, rather than standard two-strain recombinant inbred strains, is that together the CC archives over 90% of the allelic diversity of the entire mouse species (Roberts et al., 2007). Over 170,000 SNPs have been typed in each CC strain and their genotypes have been imputed at over 600,000 SNPs (Mott et al., 2000; Yalcin et al., 2005; Collaborative Cross Consortium, 2012; Zhang et al., 2014). Due to its low long range linkage disequilibrium, CC gene mapping avoids false positive discoveries that can confound conventional studies (Collaborative Cross Consortium, 2012). By archiving hundreds of recombination events and with all genomic sequences available, the CC allows mapping of loci with unprecedented accuracy (Ram et al., 2014; Kristic et al., 2018).

The MexTAg transgenic mouse is a well-characterised mouse model of asbestos-induced mesothelioma. In MexTAg mice, expression of the oncogenic simian virus 40 large T antigen (SV40 TAg) is directed to mesothelial cells by use of the cell type specific mesothelin promoter. In mice carrying a high copy number of TAg, mesothelioma is fully penetrant after asbestos exposure; in mice carrying a lower copy number, 85% of mice die with mesothelioma. This compares to an incidence of around 30% in asbestos exposed wild type mice (Robinson et al., 2006). Expression analysis comparing MexTAg and wild type mesotheliomas with their counterpart normal mesothelial cells demonstrates overlapping gene expression profiles (Robinson et al., 2015) that suggest the SV40 TAg oncogene does not affect the overall mechanism of mesothelioma development, but rather it phenocopies p16 loss and as a consequence, onset of disease is also more rapid relative to wild type mice (around 20 weeks after asbestos exposure). Notably, while mesothelioma development in MexTAg mice is uniform, predicable and occurs with similar pathology to human tumours (Robinson et al., 2011), it only occurs after asbestos exposure. Importantly, unlike previous human studies, the use of a mouse model allows for the control of specific environmental/lifestyle variables such as the degree of asbestos exposure and diet.

Here, we combine the CC and MexTAg resources to demonstrate for the first time the capacity of host genetics to influence asbestos related disease phenotypes. This proof of principle study confirms the feasibility of this novel approach and provides a rational framework required for identification of multiple low risk gene variants that has so far eluded conventional human mesothelioma genetic studies.

## 2 Methods and materials

### 2.1 Mice

Collaborative Cross-MexTAg breeding (RA/3/300/106; RA/3/300/107) and experimental (RA/3/100/1,408; RA3/100/1730) protocols were approved by the University of Western Australia animal ethics committee (UWA AEC) in accordance with the Australian code for the use of animals in medical research (Council, 2013). Male CC mice were generously provided by Geniad Pty Ltd. from its colonies at the Animal Resource Centre (Perth, Western Australia). Additional male CC strains (denoted by CCXXX format, Supplementary Table S1) were obtained from the University of North Carolina CC colony. Female parental homozygous 266-MexTAg mice (C57Bl/6, H2-K^b^) were bred and housed at the University of Western Australia (UWA) Biomedical Research Facility (Perth, Western Australia).

### 2.2 Asbestos exposure experiments

All CCMT progeny and 266-MexTAg heterozygous control mice received a total of 6 mg asbestos administered as two intraperitoneal (i.p.) injections of 3 mg sterile asbestos (IUCC reference sample; Wittenoom Gorge crocidolite) suspended in 0.5 mL PBS at weeks 0 and 4 and survival calculated from the day of first injection as per published protocols (Robinson et al., 2006; Robinson et al., 2011). All mice were humanely euthanased in accordance with the UWA AEC approved animal care services standard operating procedure “*201–8 Euthanasia of the Mouse*”, primarily via methoxyflurane inhalation overdose (Medical Developments International, Australia. Penthrox^®^ methoxyflurane, 99.9% v/v) followed by cervical dislocation.

### 2.3 Tissue collection and histology

Tissue samples including macroscopic tumour, spleen, kidney, liver, and diaphragm were collected for histological and genetic analysis (RNAlater, QIAEN). Cell lines were generated from peritoneal ascitic fluid and macroscopic tumour when possible (see below). Tissues were fixed in 4% formaldehyde (Amber Scientific Pty Ltd., Perth, Western Australia) for 24–48 h, preserved in 70% ethanol prior to embedding in paraffin blocks (Surgipath Paraplast paraffin Leica Biosystems, Australia). Five micrometre (5 µm) sections were cut and stained with haematoxylin and eosin (H&E). Histopathological analysis was performed via bright field microscopy (Nikon Eclipse E200 microscope (Minato City, Tokyo, Japan), with selected sections scanned using Leica (Aperio) Scanscope Digital Slide Scanner. Ten (10) histological features were reviewed with each feature defined as representative of either benign or malignant disease. Additionally, the histological subtype of each mouse was also noted (epithelioid, sarcomatoid or biphasic).

### 2.4 Cell culture: establishing ascites and tumour derived CCMT cell lines

Ascites was collected under aseptic conditions immediately post euthanasia and placed in tissue culture flasks with at least 2X volume of DMEM (Sigma) supplemented with 15% NCS (HyClone Cytiva), under 5% CO_2_ and 95% humidity. Likewise, when possible, solid tumours were dissociated using scalpels and small fragments cultured as described above. Cells were passaged as required and frozen stocks stored in liquid nitrogen.

### 2.5 Statistical analysis

Kaplan–Meier survival curves were analysed by log rank test (Mantel-Cox) with >95% confidence intervals (CI). Correlations were analysed by Pearson’s test for correlation with 95% CI. Statistical analyses were performed using Graph Pad Prism V8.4.2 (Graph Pad Software Inc., United States of America). One-way ANOVA test for variance was used to analyse data from three or more groups. The non-parametric, unpaired, two-tailed *t*-test was used to compare data from two test groups. Frequencies of histological features between groups was compared using Chi-Square frequency table analysis and logistic regression. Endpoints for survival analyses included disease latency; disease progression; overall survival (OS) and cancer-specific survival (CSS), defined as time from first injection until death from mesothelioma. Disease latency and disease progression were analysed using linear regression. OS and CSS were analysed using Cox proportional hazard modelling. Multivariate analysis was performed using stepwise backward selection with all significant variables in univariate analysis included in the starting model with a *p* < 0.05 required to remain in the model. Variables were considered significant at α < 0.05. Features with less than 5% of a feature either present or not present across the cohort were excluded to reduce type 1 error. For histological analyses all statistical analyses were performed in SAS v9.4 (IBM; Cary, NC, United States).

### 2.6 Identification of candidate modifier genes (GeneMiner analyses)

Candidate modifier genes were identified using the GeneMiner bioinformatic suite (https://www.sysgen.org/Geniad2/) that combines the HAPPY (Mott et al., 2000; Durrant et al., 2011) and DO-QTL programs to define the founder haplotypes associated with respective biological traits as previously described (Ram et al., 2014). Genome wide scans were performed to define chromosomal locations of peak SNPs associated with each respective phenotype, such as overall survival, disease progression, latency, and ascites volume. Multinomial logistic regression models were fitted for each trait at each locus and ANOVA chi-square tests used to estimate the *p*-value of association. A false discovery rate (FDR) of *p* < 0.001 was used to define significant genome-wide linkage (Benjamini et al., 2001). The founder strain(s) contributing to each trait were determined by deriving coefficients (log odds ratio) of the fit from the multinominal regression model, as implemented in DO-QTL.

Chromosomal regions containing peak eQTL were interrogated using either the Mouse Genome Informatics database (https://www.informatics.jax.org/) or Sanger Sequencing database (via the GeneMiner informatics portal; https://www.sysgen.org/Geniad2/) to identify candidate modifier genes.

### 2.7 Interrogation of human mesothelioma datasets

RNAseq datasets from the Bueno (Bueno et al., 2016) and The Cancer Genome Atlas (TCGA-MESO) human mesothelioma cohorts were used to investigate the association between the expression of candidate modifier genes in tumours and age of mesothelioma patients at the time of diagnosis/surgery. To utilize RNAseq datasets from the Bueno cohort, we imported RNAseq datasets in raw FASQ file format from the European genome-phenome archive (EGA, accession code: EGAS00001001563) and aligned them with Kallisto (v0.46.1) against the human reference genome (GRCh38). The “TCGAbiolinks” R package was used to retrieve RNAseq data from TCGA datasets in the STAR aligned raw gene count format from the Genomic Data Commons (GDC) portal. We converted CCMT candidate modifier gene symbols into homologous human gene symbols and performed univariate cox regression analysis using the “coxph” function from the “survival” R package to identify genes associated with age at the time of surgery, and age at the time of diagnosis in the Bueno and TCGA cohorts respectively. Genes with *p*-value < 0.05 were selected for Kaplan Meier survival analysis using the “survminer” R package. All analyses were conducted using Rstudio (version 4.1.0).

## 3 Results

### 3.1 Generation of collaborative Cross-MexTAg (CCMT) mice

**FIGURE 1:**
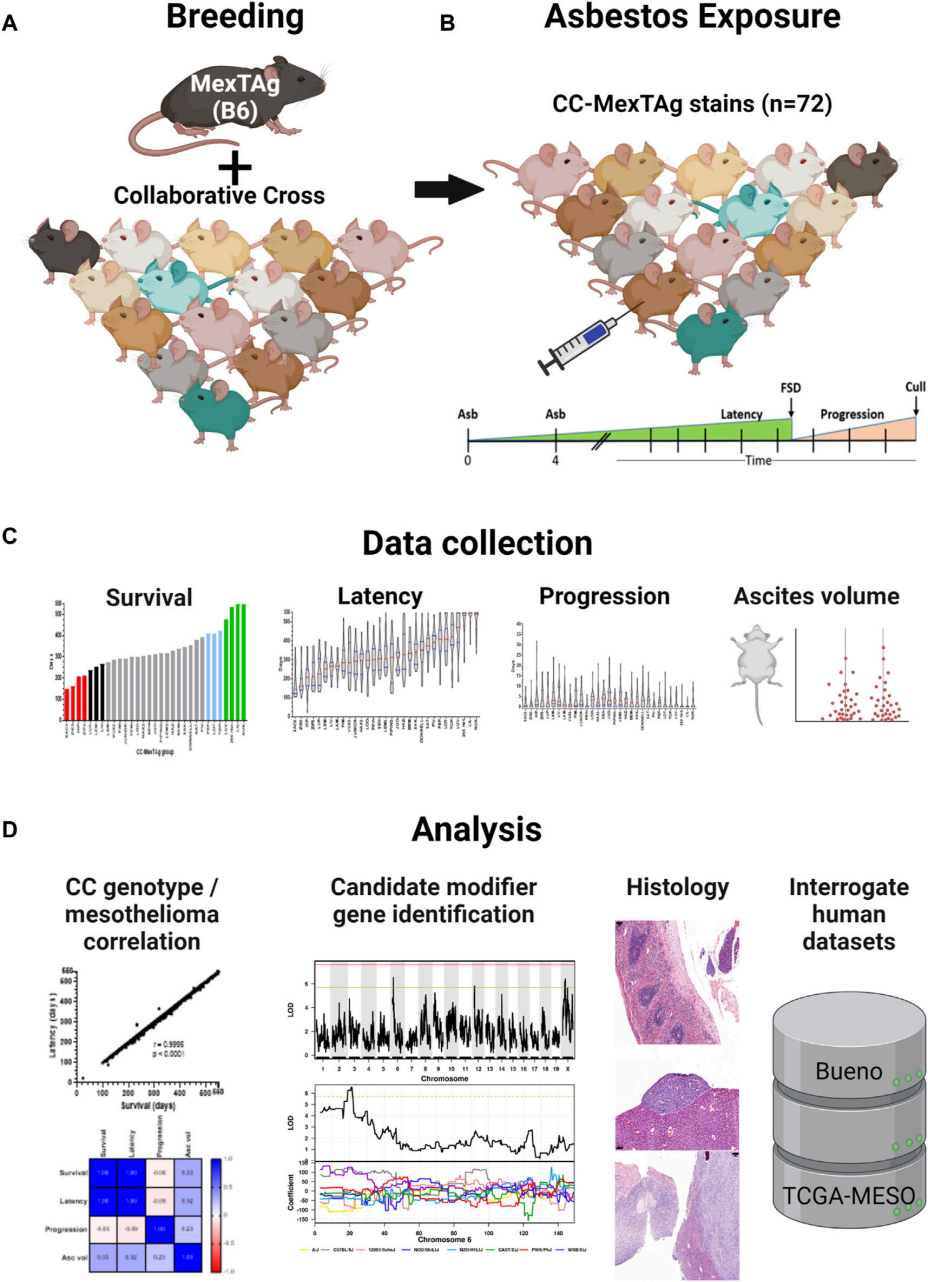
CC-MexTAg (CCMT) experimental schema: **(A)** Female 266Hom MexTAg mice were crossed with male mice from 71 distinct collaborative cross (CC) strains and, **(B)** the resultant CCMT progeny (n = 2,565 mice total) exposed to crocidolite asbestos via two intraperitoneal injections (6 mg total) 4 weeks apart. Mice were assessed for asbestos related disease (ARD) development and culled at predefined welfare endpoints or at a maximum of 18 months (548 days) after first asbestos exposure, whichever occurred first. **(C)** Phenotypic data were collected on overall survival, asbestos related disease latency/progression and ascites volume. **(D)** Asbestos related disease was confirmed on histology and correlative analyses on phenotypic traits performed. Figure created with BioRender.com.

The generation of MexTAg mice has been described previously (Robinson et al., 2006; Robinson et al., 2011). To generate CCMT mice, female homozygous 266-MexTAg mice, that contain two copies of the SV40 TAg transgene, were crossed with male mice from 71 distinct CC strains in a sequential, staggered, batch-breeding protocol (Figure 1A). All CC-MexTAg progeny carried a single copy of the SV40 TAg transgene but differ genetically based on the genotype of the parental CC strain. All attempts were made to match CCMT experimental groups for age and gender balance prior to asbestos exposure. Characteristics of CCMT groups are described in Supplementary Table S1.

### 3.2 Asbestos induced mesothelioma

Seventy-one (71) groups of CCMT mice and a single group of 266-MexTAg heterozygous controls (266Het, containing a single copy of TAg; cohort median n = 37/group, range 18–45/group, 2,565 total mice) received intraperitoneal asbestos injections as previously described (Robinson et al., 2006; Robinson et al., 2011) and were monitored for ARD development (Figure 1B). Asbestos-exposed mice were assessed for traits including overall survival (time from first asbestos exposure to cull); ARD latency (time from first asbestos exposure to first signs of disease; FSD); ARD progression (time from FSD to euthanasia) and mean ascites volume (Figure 1C, Supplementary Table S2). Mice were culled at predefined welfare endpoints (namely, presence of ascites or loss of condition), or at the 18 months (548 days) experimental endpoint, whichever occurred first. Tissue samples were collected upon euthanasia and analyses of phenotypic parameters and histological sections performed (Figure 1D).

### 3.3 Asbestos exposed CCMT mice develop asbestos related disease and display a three-fold difference in overall survival

**FIGURE 2:**
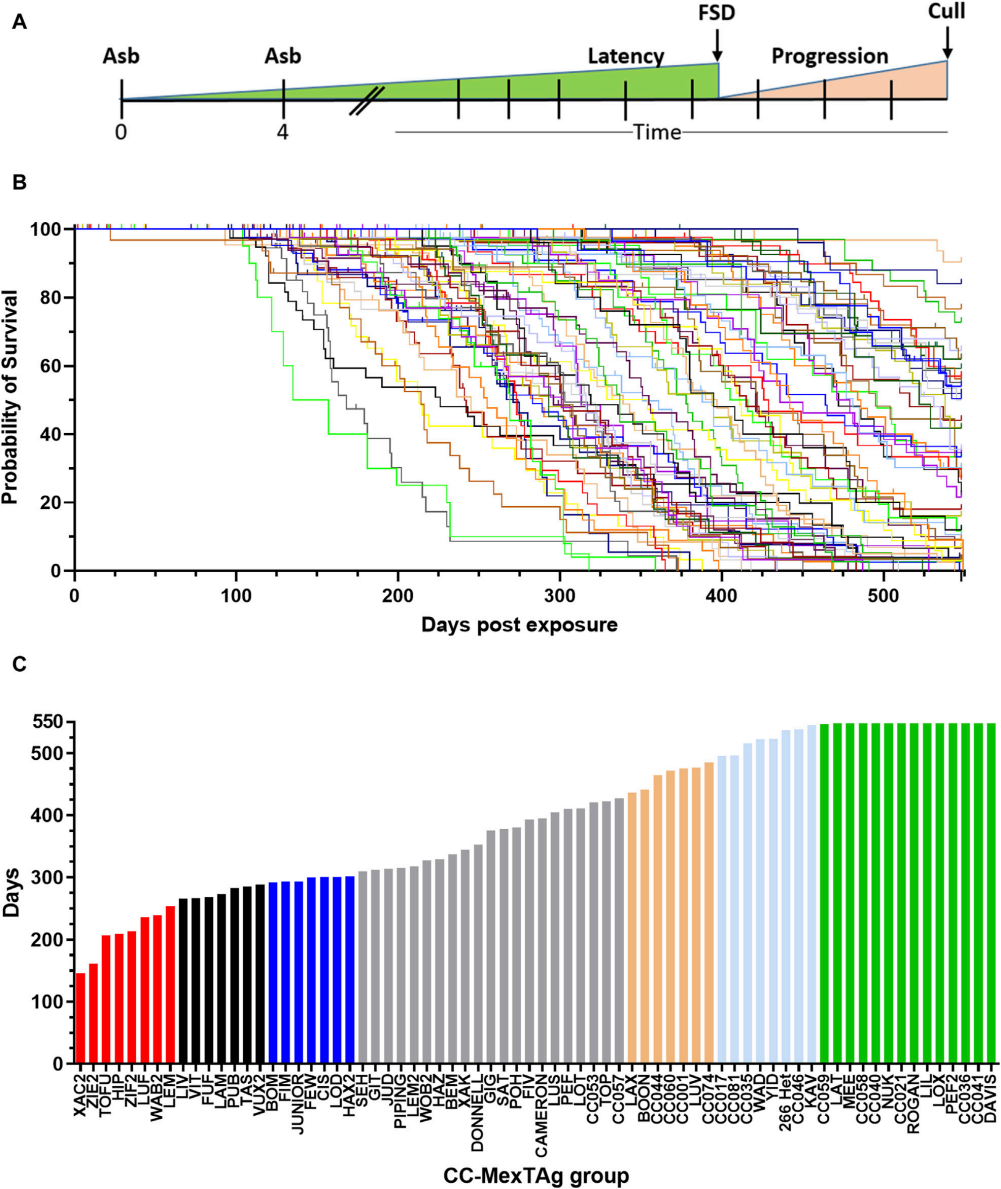
Variable overall survival in asbestos exposed CCMT mice. 71 groups of CCMT mice and the heterozygous parental 266-MexTAg (266-Het) control group were exposed to asbestos and monitored for asbestos related disease (ARD) development over 18 months (548 days). **(A)** Experimental schematic, **(B)** Kaplan-Myer plot (each line represents a CCMT or MexTAg (B6) control group) and **(C)** ranked median survival data demonstrating a 3.75-fold difference in overall median survival between asbestos exposed CCMT groups. **(B,C)** Data shows asbestos related disease associated median survival (days from first asbestos exposure to cull, n = 2,245 mice. Non-ARD deaths have been censored). For ranked median survival **(C)** percentiles indicated by colour; red ≤10%, black 11%–20%, blue 21%–30%, gold = 70–79%, light blue = 80–89% and green ³ 90%. FSD = first signs of disease.

To assess the impact of host genetics on ARD development, overall survival (time from first asbestos exposure to cull) was analysed (Figures 2A, B). Significant variation in overall survival was observed between different asbestos exposed groups (*p* > 0.001), with a 3.75-fold difference observed between groups with the shortest, compared to those with the longest median survival (Figure 2C; red and green bars respectively). ARD incidence was consistent across most CCMT groups (cohort median 87%, range 50%–100%, with only progeny of four CC strains (SEH 50%; LIV 62.5%; ZIE2 66.7% and LUF 67.5%) having an ARD incidence more than two-standard deviations lower than the cohort mean (87% ± 18.4% (2SD), Supplementary Table S1). Interestingly, heterozygous parental 266-MexTAg control mice, which contain a single copy of the SV40 TAg transgene, displayed similar median survival (536.5 days) and incidence (93.8%) to the longest surviving CCMT groups (groups CC059 to DAVIS green bars Figure 2C; 548 days; 87.5% ± 5% respectively, Supplementary Table S1); suggesting that a) the B6 strain (one of the CC founders and host of the MexTAg transgene) confers more protection than the other founders; and b) such modifiers are not linked to the single copy of TAg as linked genes do not mask the influence of host genetics on ARD development.

### 3.4 Variation in survival is determined by disease latency

**FIGURE 3:**
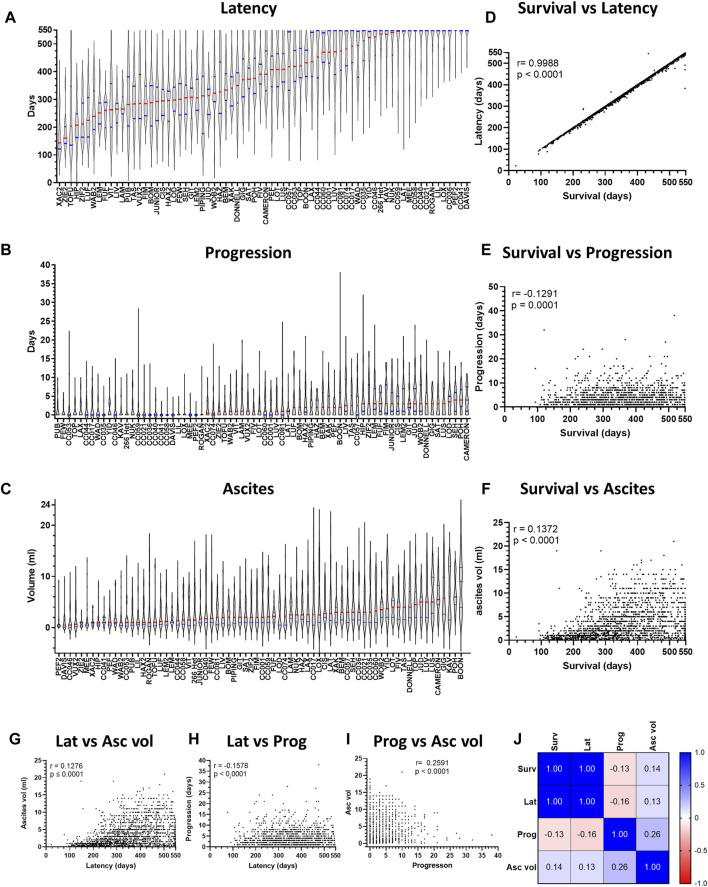
Variation in overall survival is revealed during ARD latency, but not progression. Violin plots depicting variation in disease **(A)** latency and **(B)** progression and **(C)** ascites volume for asbestos exposed CCMT groups. Data ranked by Red bars = median. Blue bars = quartiles. **(D–F)** Plots depict correlation between overall survival (*x*-axis) and ARD latency, progression, and ascites volume respectively. **(G–I)** Plots depict correlation between respective phenotypic traits. For plots D to I, each dot represents an individual mouse with ARD. **(J)** Heat map correlation matrix for respective phenotypic traits. r = Pearson’s coefficient. Asc vol = ascites volume, Lat = latency, Prog = progression, Surv = survival.

We next assessed variation in ARD latency (time from first asbestos exposure to FSD) and progression (time from FSD to Cull; Figures 3A, B). The variation in ARD latency was identical to that observed for overall survival (Pearson’s correlation coefficient r = 0.9988), while an inverse correlation was observed between overall survival and disease progression (r = −0.1291, Figures 3D, E). The implications of the strong positive correlation between overall survival and disease latency are two-fold: firstly, it suggests that overall survival and disease latency are likely the same trait and secondly, it indicates that the variation observed in overall survival occurred during the latency period and not once disease was established (i.e., during ARD progression).

### 3.5 Variation in host genetics affects asbestos related disease phenotypes

We further assessed the relationship between each of the different ARD phenotypic traits (Figures 3C–I). Consistent with the strong positive correlation between latency and survival, both survival and latency demonstrated a positive correlation with mean ascites volume (Figures 3F, G). However, little correlation was observed between ARD progression and all other phenotypes (Figures 3H–J). Taken together, these data demonstrate that asbestos-exposed mice developed ARD during a long latency period, consistent with the observed long period for human mesothelioma development.

### 3.6 Asbestos exposed CC-MexTAg mice develop histological features characteristic of human mesothelioma

**FIGURE 4:**
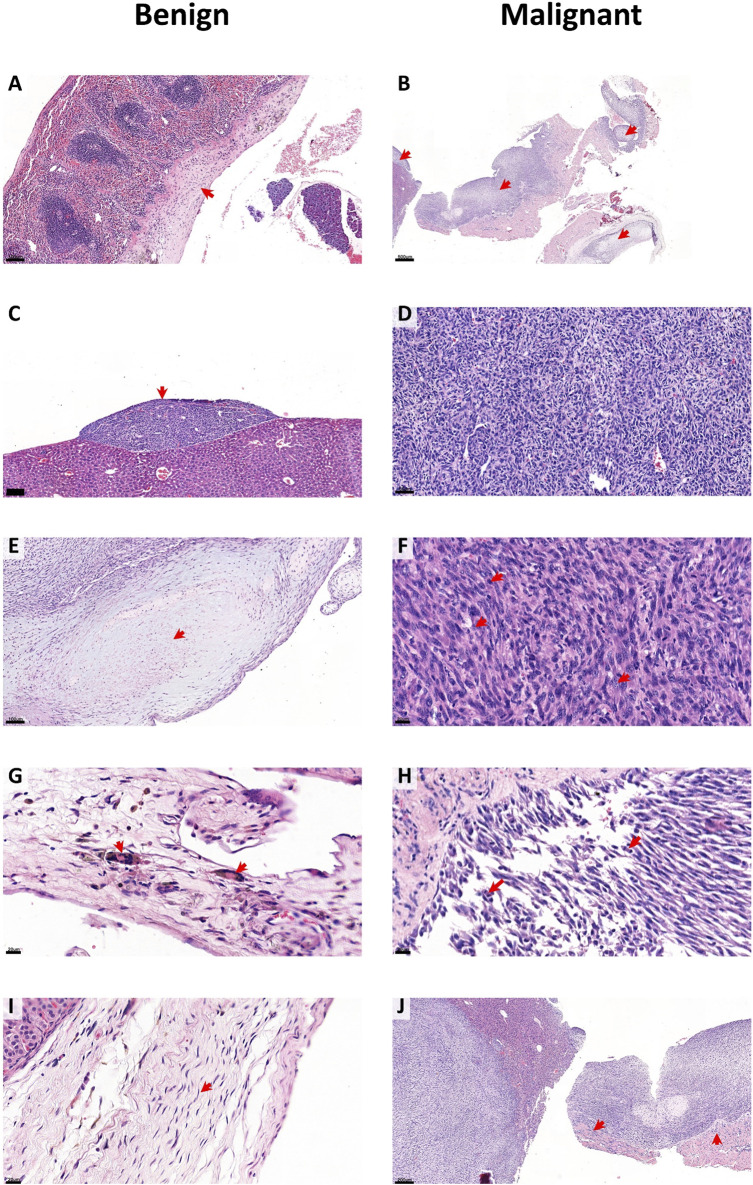
CCMT asbestos related disease histology. Representative images of benign **(A,C,E,G,I)** and malignant **(B,D,F,H,J)** features (red arrows) from histological assessment of 403 asbestos exposed CCMT mice. Benign features include **(A)** thickening, **(C)** plaque formation, **(E)** paucicellular regions, **(G)** giant cell presence and **(I)** regular nuclei. Malignant features include **(B)** overt tumour, **(D)** hypercellularity, **(F)** nuclear atypia (multiple nucleoli, coarse chromatin), **(H)** mitotic figures and **(J)** invasion. Scale bars; 20 µm **(F,G,H,I)**; 50 µm **(D)**; 100 µm **(A,C,E)**; 200 µm **(J)** and 500 µm **(B)**. Overt tumour **(B,J)** is of sarcomatoid subtype.

We next sought to characterise the histological features associated with asbestos exposed CCMT mice. Spleen, kidney, liver, and diaphragm were harvested from all asbestos-exposed mice at predefined disease-associated or experimental endpoints and were assessed for signs of histological disease. Bulk tumour tissue was also assessed when available. Histological review for benign and malignant features was performed on sections from a subset of 403 individual mice from 12 distinct CCMT groups representing either short (XAC2, ZIE2, HIP, ZIF2, LUF and LEM), medium (LOT, TOP, LUV, 266-Het) or long (NUK and LIL) survival groups, based on each respective groups’ median survival time (Figure 2C: “short” and “long” groups represent either the lowest 30% or highest 30% survival percentile respectively). Histological features representative of benign changes included mesothelial thickening, plaque development, paucicellularity within mesothelial layers, presence of giant cells and regular nuclei without atypia. Representative malignant histological changes included overt tumour (100% sarcomatoid), hypercellularity, nuclear atypia (multiple nucleoli, coarse chromatin), the presence of mitotic figures and invasion of surrounding tissue (Figure 4).

A range of benign/fibrotic and neoplastic histological features was observed in all asbestos-exposed mice. Frequency table analysis and logistic regression used to compare histologically features between short and long survival groups indicated that CCMT mice whose samples contained plaques (*p* = 0.004), regular nuclei (*p* = 0.019), mitotic figures (*p* < 0.001) and areas of invasion (*p* = 0.022; Table 1) were significantly associated with short survival. No significant differences between short and long survival groups were found for thickening, presence of giant cells, overt tumour, or regions of hypercellularity (Table 1).

**TABLE 1:** Histological features in short vs. long survival groups. Variables with significant differences between groups highlighted in bold.

Feature		Short survival	Long survival	χ2 p
Thickening, n (%)	Absent	0 (0%)	0 (0%)	-
	Present	226 (56%)	177 (44%)	
Plaque, n (%)	Absent	4 (1%)	15 (4%)	**0.004**
	Present	222 (55%)	162 (40%)	
Giant Cells, n (%)	Absent	190 (47%)	147 (37%)	0.783
	Present	36 (9%)	30 (7%)	
Paucicellular, n (%)	Absent	175 (43%)	101 (25%)	**<0.001**
	Present	51 (13%)	76 (19%)	
Regular Nuclei, n (%)	Absent	5 (1%)	13 (3%)	**0.019**
	Present	221 (55%)	164 (41%)	
Overt Tumour, n (%)	Absent	131 (33%)	86 (21%)	0.061
	Present	95 (24%)	91 (23%)	
Hypercellularity, n (%)	Absent	5 (1%)	1 (0%)	0.209
	Present	221 (55%)	176 (44%)	
Nuclear Atypia, n (%)	Absent	1 (0%)	1 (0%)	0.863
	Present	225 (56%)	176 (44%)	
Mitotic Figures, n (%)	Absent	30 (7%)	50 (12%)	**<0.001**
	Present	196 (49%)	127 (32%)	
Invasion, n (%)	Absent	66 (16%)	34 (8%)	**0.022**
	Present	160 (40%)	143 (36%)	

Wald Chi-square *p*-values presented.

### 3.7 Distinct histological features associated with overall survival revealed during disease latency

Consistent with our previous observations of phenotypic traits, univariate analysis revealed no histological variables that were significantly associated with disease progression (Supplementary Table S3). Conversely, for disease latency, the presence of plaques (*p* = 0.033) and regular nuclei (*p* = 0.015) were associated with groups with shorter latency, while regions of paucicellularity in the mesothelial layer (*p* = 0.002), hypercellularity (*p* = 0.038) and invasion (*p* = 0.012) were associated with longer disease latency (Table 2). Multivariate analysis revealed that presence of plaques was independently associated with shorter disease latency time (adjusted *p* = 0.035), whereas the presence of paucicellular regions (adjusted *p* = 0.003) and evidence of invasion (adjusted *p* = 0.016) were independently associated with longer disease latency (Table 2).

**TABLE 2:** Univariate and multivariate analysis results for latency time using general linear regression. Significant values highlighted bold.

Feature		Univariate analysis				Multivariate analysis			
		Mean time (Months)	95%CI		*p*	Adjusted mean time (Months)	Adjusted 95% CI		*p*
Thickening	Absent				-				-
	Present	11.22	10.73	11.71					
Plaque	Absent	13.62	11.37	15.88	**0.033**	13.49	11.26	15.72	**0.035**
	Present	11.10	10.60	11.60		11.04	10.43	11.65	
Paucicellular	Absent	10.68	10.09	11.27	**0.002**	11.49	10.27	12.71	**0.003**
	Present	12.39	11.52	13.25		13.05	11.70	14.40	
Giant cells	Absent	11.30	10.77	11.84	0.439				-
	Present	10.78	9.56	12.00					
Regular nuclei	Absent	14.04	11.72	16.35	**0.015**				-
	Present	11.09	10.59	11.59					
Overt tumour	Absent	10.77	10.10	11.44	0.055				-
	Present	11.74	11.02	12.46					
Hypercellularity	Absent	6.99	2.97	11.01	**0.038**				-
	Present	11.28	10.79	11.78					
Nuclear atypia	Absent	10.26	3.26	17.26	0.788				-
	Present	11.22	10.73	11.72					
Mitotic figures	Absent	12.13	11.02	13.23	0.072				-
	Present	10.99	10.45	11.54					
Invasion	Absent	10.13	9.14	11.11	**0.012**	11.58	10.17	12.98	**0.016**
	Present	11.58	11.02	12.14		12.96	11.77	14.14	

### 3.8 Paucicellularity and hypercellularity associated with improved overall and cancer-specific survival

Cox proportional hazards regression was used to analyse the overall (OS) and cancer-specific survival (CSS) data from individual asbestos exposed animals. Mice that survived until the end of the study (18 months) were censored for both OS and CSS.

Features found to be good prognostic factors for OS included paucicellular regions (HR 0.66; 95%CI 0.52–0.84; *p* < 0.001) and regions of hypercellularity (HR 0.31; 95%CI 0.14–0.70; *p* = 0.005). Regions of regular nuclei (HR 2.19; 95%CI 1.17–4.11; *p* = 0.015) and mitotic figures (HR 1.51; 95%CI 1.13–2.02; *p* = 0.005) were poor prognostic factors (Table 3). In multivariate analysis only paucicellularity was independently associated with overall survival with a 44% reduction in the hazard of death from any cause (Table 3). Similarly for cancer specific survival, paucicellularity (HR 0.65; 95% CI 0.51–0.84; *p* = 0.001) and hypercellularity (HR 0.25; 95% CI 0.11–0.57; *p* = 0.001) were considered good prognostic factors, whereas regular nuclei (HR 2.47; 95% CI 1.22–4.99; *p* = 0.012) and mitotic figures (HR 1.87; 95% CI 1.35–2.59; *p* < 0.001) were significant poor prognostic factors in univariate analysis (Table 4). Paucicellularity (adj. HR 0.74; 95% CI 0.57–0.97; adj. p = 0.027) and the presence of mitotic figures (adj. HR 1.68; 95%CI 1.94–2.36; adj. p = 0.003) were independently associated with improved cancer specific survival in multivariate analysis (Table 4).

**TABLE 3:** Overall survival analysis using Cox regression modelling results for each variable in univariate analysis and multivariate analysis. Significant variables highlighted bold.

Feature	Univariate analysis				Multivariate analysis			
	Hazard ratio	95%CI		*p*	Adj. Hazard ratio	Adjusted 95% CI		*p*
Thickening				-				-
Plaque	1.53	0.90	2.61	0.120				-
Paucicellular	0.66	0.52	0.84	**<0.001**	0.66	0.52	0.84	**<0.001**
Giant Cells	0.98	0.73	1.32	0.903				-
Regular Nuclei	2.19	1.17	4.11	**0.015**				-
Overt Tumour	0.90	0.73	1.12	0.356				-
Hypercellularity	0.31	0.14	0.70	**0.005**				-
Nuclear Atypia	1.75	0.25	12.46	0.577				-
Mitotic Figures	1.51	1.13	2.02	**0.005**				0.056
Invasion	0.80	0.63	1.03	0.079				-

Adj. adjusted.

**TABLE 4:** Cancer-specific survival analysis using Cox regression modelling results for each variable in univariate analysis and multivariate analysis. Significant variables highlighted bold.

Feature	Univariate analysis				Multivariate analysis			
	Hazard ratio	95%CI		p	Adj. Hazard ratio	Adjusted 95% CI		p
Thickening				-				-
Plaque	1.75	0.96	3.20	0.068				-
Paucicellular	0.65	0.51	0.84	**0.001**	0.74	0.57	0.97	**0.027**
Giant Cells	0.86	0.61	1.20	0.361				-
Regular Nuclei	2.47	1.22	4.99	**0.012**				-
Overt Tumour	0.93	0.74	1.16	0.503				-
Hypercellularity	0.25	0.11	0.57	**0.001**				-
Nuclear Atypia	-	-	-	0.970				-
Mitotic Figures	1.87	1.35	2.59	**<0.001**	1.68	1.19	2.36	**0.003**
Invasion	0.93	0.70	1.22	0.581				-

Adj. adjusted.

Taken together the histological data from a subset of asbestos exposed CCMT mice indicates that mice develop many of the ARD related histological features that are characteristic of human mesothelioma.

### 3.9 Candidate modifier genes associated with ARD phenotypic traits

**FIGURE 5:**
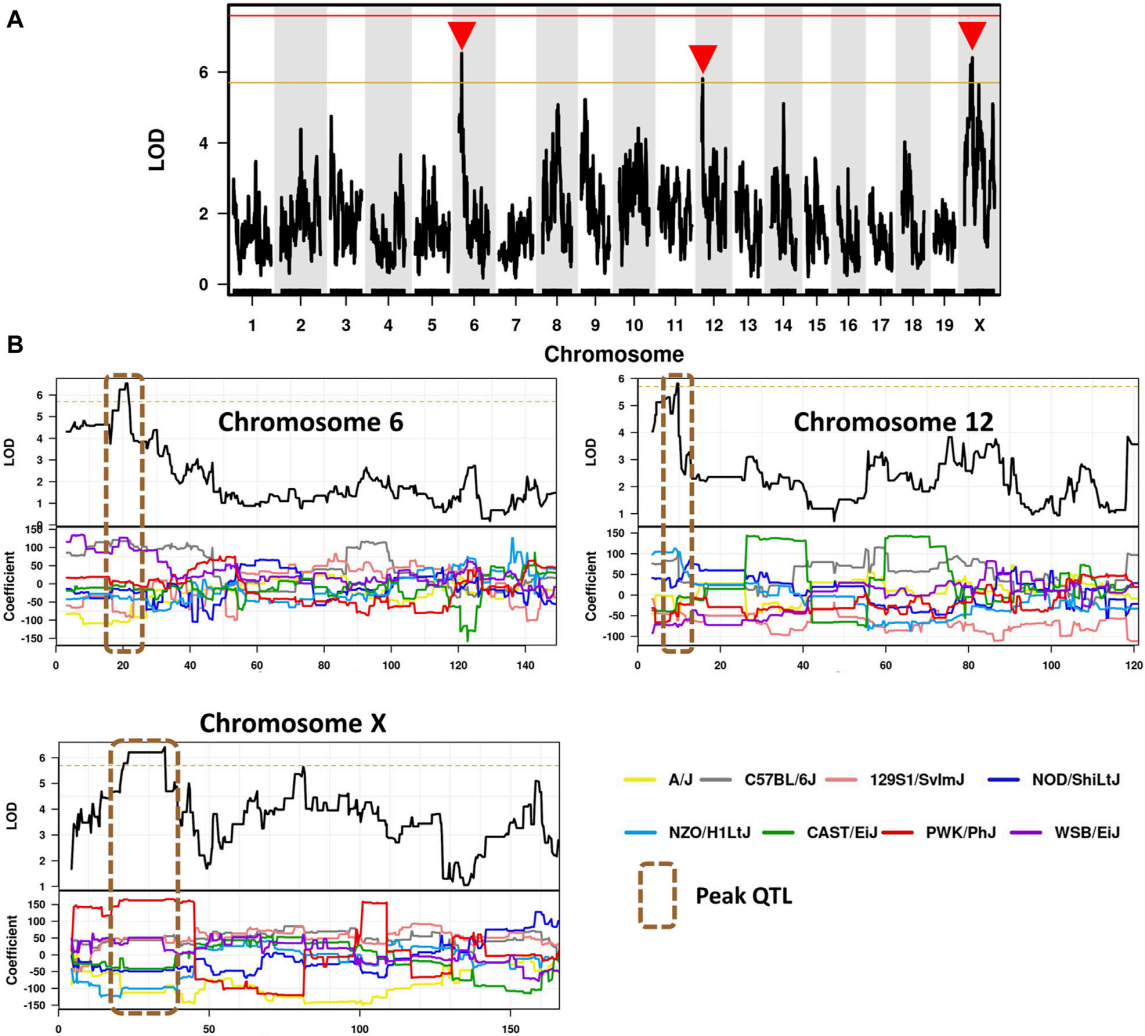
Identification of CCMT candidate modifier genes. **(A)** Genome-wide scan based on median survival/latency in 71 asbestos exposed CCMT strains depicting highly suggestive QTL on chromosomes 6, 12 and X (red arrows). Genotyping, construction of CC strain haplotypes, and linkage analysis were performed as previously described (Ram et al., 2014). Phenotype data was analysed using the GeneMiner MugaQTL(as is) setting. The *x*-axis shows the chromosomal position, and the *y*-axis shows the 2log10(P) values; the *p*-values were derived from the linkage haplotype data. **(B)** Top: Plot of LOD scores along chromosomes 6, 12 and X. Bottom: Founder allele coefficients: plot of the calculated log-odds ratio of eight founder alleles over the chromosome where the founders are color-coded. Dotted line highlights chromosomal region containing peak QTL.

To identify candidate modifier genes associated with asbestos related traits, we used the GeneMiner bioinformatic portal. For each trait, multinomial logistic regression models were fitted at each locus and ANOVA chi-square tests used to estimate the *p*-value of association. A summary of candidate modifier genes associated with each phenotypic trait is provided in Figure 5; Table 5. With respect to overall survival/latency as traits, we identified major effect QTL (LOD ≥5.8, founder coefficient range −100 to +150) on chromosomes 6, 12, and X (Figure 5A), with numerous protein-coding genes located around each peak QTL (Table 5; Supplementary Table S4). Similarly, eight additional QTL located on chromosomes 2, 3, 4 (two QTL), 5, 9, 16 and X were identified when using ascites volume as the trait; although the founder coefficient range was considerably smaller (−1 to +1.5), suggesting each of these genes alone would only have a minimal influence on ascites volume (Table 5; Supplementary Table S5; Supplementary Figure S1).

**TABLE 5:** GeneMiner analysis summary data highlighting the location, effect size (founder coefficient) and number of known QTL and protein coding gens located at peak QTL for respective CCMT ARD phenotypes.

Trait/Phenotype	Peak QTL					Known QTL		Protein genes	
	Chromo-some	LOD score	Position (Mb)	CC founder haplotype	Founder coefficient	@ peak QTL	QTL range	@ peak QTL	QTL range	
			(Range, Mb)							
Survival/Latency	6	6.6	21.249	(+) B6, WSB	100+	1	10	7	19	
			(17–22)	(−) A/J, 129S	−100					
	12	5.8	9.855	(+) NZO, B6	100+	2	7	6	15	
			(7–10)	WSB (−)	−100					
	X	6.4	35.239	(+) PWK	150+	0	0	25	63	
			(20–40)	(−) A/J, NZO	−100					
Ascites volume	2	6.4	107.369	(+) NZO	1+	0	12	1	39	
			(103.7–108.61)	(−) CAST/129S1	−1					
	3	6.3	118.2–119.3	(+) B6	0.5+	1	4	2	29	
			(116.3–121.2)	(−) NZO	−0.75					
	4	6.4	118.0–118.5	(+) CAST	0.5+	14	32	63	187	
			(108.2–119.1)	(−) PWK	−0.75					
	4	6.4	132.1–132.5	(+) WSB	0.75+	9	17	67	111	
			(130.3–132.6)	(−) PWK	−0.75					
	5	5.9	17.5–17.8	(+) NOD	1.5+	2	5	9	24	
			(15.2–20.3)	(−) CAST	−0.5					
	9	6.6	122.63	(+) NZO/129S1	0.9+	2	5	16	51	
			(122–123.88)	(−) A/J	−0.9					
	16	5.9	84.25	(+) NOD/WSB/129S1	0.7+	3	11	7	13	
			(78–85)	(−) A/J	−0.7					
	X	5.8	137.762	(+) 129S1	0.7+	2	2	14	17	
			(137–138)	(−) B6	−1					
Progression	Nil	**-**	**-**	**-**	**-**	**-**	**-**	**-**	**-**	

ARD, asbestos related disease; LOD, logarithm of odds; QTL, qualitative trait loci.

Of the 97 CCMT candidate modifier genes identified across the three major effect QTL for ARD survival/latency, four occurred at the peak QTL on chromosome 6 (*Kcnd2, Tspan12, Ing3, Cped1*); six on chromosome 12 (*Matn3, Wdr35, Ttc32, Osr1, Nt5c1b, Rdh14*); and 17 on the X chromosome (*Il13ra1, Pgrmc1, Septin6, Ndufa1, Nkrf, Steep1, Akap17b, Zcchc12, Lonrf3, Slc25a5, Ct47, Gm14819, Rhox1, Gm14569, Gm10486, Akap14, Dock11;* Figure 5B, Table 5; Supplementary Table S4). While no individual candidate modifier gene was observed to have a significant influence over any other gene at each respective QTL, many are known to be associated with advanced or metastatic cancers (Supplementary Table S4), suggesting a complex polygenic interplay between host genes affects ARD survival/latency.

### 3.10 Human homologues of CCMT candidate modifier genes impact outcome in human mesothelioma

**FIGURE 6:**
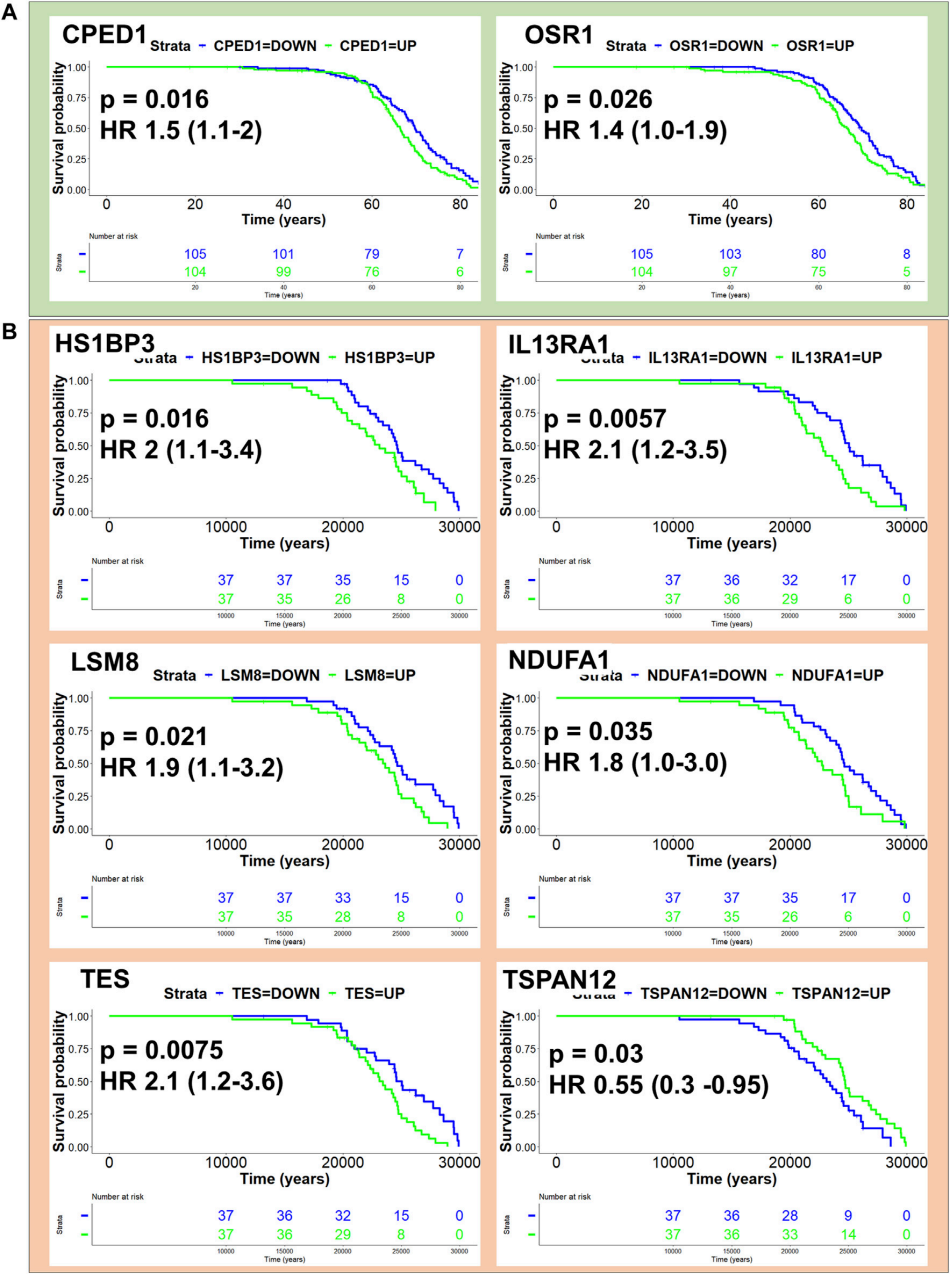
Homologues of CCMT candidate modifier genes influence human mesothelioma. Kaplan Myer plots depicting human homologues of CCMT candidate modifier genes with *p*-value < 0.05 and their effect on patient outcome in **(A)** Bueno (green) and **(B)** TCGA mesothelioma cohorts. KM survival analysis performed using the “survminer” R package.

We next sought to assess the potential for CCMT candidate modifier genes to influence disease outcome in human mesothelioma. All CCMT candidate modifier genes spanning the major effect QTL were converted into homologous human gene symbols and univariate Cox regression analysis performed on human mesothelioma RNAseq datasets to identify genes associated with age at the time of surgery and age at the time of diagnosis in the Bueno (Bueno et al., 2016) and TCGA-MESO cohorts, respectively. Expression of two candidate genes, odd-skipped related 1 (*OSR1*, HR 1.4, *p* = 0.026) and cadherin-like and PC-esterase domain containing 1 (*CPED1*; HR 1.5, *p* = 0.016), were significantly associated with a poorer outcome in the Bueno cohort (Figure 6A; Supplementary Table S6). Likewise, expression of five of the six genes identified in the TCGA cohort (Figure 6B; Supplementary Table S7), namely, HCLS1 binding protein 3 (*HS1BP3*, HR 2, *p* = 0.016); interleukin 13 receptor alpha 1 (*IL13RA1*; HR 2.1, *p* = 0.0057); LSM8 homolog, U6 small nuclear RNA associated (*LSM8*, HR 1.9, *p* = 0.021); NADH: ubiquinone oxidoreductase subunit A1 (*NDUFA1*, HR 1.8, *p* = 0.35) and testin LIM domain protein (*TES*, HR 2.1, *p* = 0.0075) were significantly associated with poorer outcome. Only expression of tetraspanin 12 (*TSPAN12*, HR 0.55, *p* = 0.03) was associated with improved outcome in the TCGA-MESO dataset (Figure 6B; Supplementary Table S7). No human homologues of CCMT candidate modifier genes were common to both cohorts.

## 4 Discussion

Mesothelioma is one of a small group of cancers with a clear and almost invariable link to an aetiological agent. Currently, asbestos-exposed individuals may be aware that they are at risk, but there is limited understanding as to the level of risk and why, despite prolonged exposure, some people do not develop asbestos related malignancies like mesothelioma. The power of conventional genetic studies such as GWAS to identify genes associated with disease traits for rare diseases can be limited, and true associations might be missed as GWAS cohorts are rarely representative of the entire human population, often limited to individuals of a particular ancestral heritage (e.g., European/Caucasian), or because disease and control cohorts are not properly matched. Most importantly, GWAS often lack sensitivity for identifying genes for complex polygenic susceptibility traits. Thus, due to a combination of intrinsic technical limitations and a limited number of cases in available cohorts, GWAS are unlikely to identify multiple interacting genes associated with the susceptibility or resistance of rare cancers such as mesothelioma.

To address these issues, we developed the MexTAg Collaborative cross; a novel mouse model designed to assess ARD development via the inclusion of a single copy of the MexTAg transgene in the presence of maximum genetic diversity derived from the parental CC stains. Asbestos-exposed CCMT mice developed ARD and displayed a wide variation in overall survival between the different CCMT groups in addition to histological features consistent with human disease. The CC has been used previously to identify genes associated with tumour development. Ferguson et al. (Ferguson et al., 2015), crossed 66 CC stains with a double transgenic mouse model of UV-induced melanoma and found great variation at all stages of melanoma development (Ferguson et al., 2015). Studying nevus formation as a phenotypic trait led to the identification of *Cdon*, a regulator of sonic hedgehog, as a key gene impacting nevi development in the context of an NRAS mutation (Chitsazan et al., 2016).

Consistent with the Ferguson study, we observed a greater than 3-fold variation in overall survival between asbestos-exposed CCMT groups that strongly correlated with the disease latency, but not disease progression, demonstrating the ability of host genetics to affect asbestos related disease development prior to disease establishment. Further analysis of survival and latency data from 71 distinct CCMT groups identified three major effect quantitative trait loci linked to ARD-associated risk alleles. Of the 97 known protein-coding genes at these loci, many of the genes located at, or spanning the peak QTL are known to be associated directly with cancer development, or with cancer associated pathways. For example, *Kcnd2, Tspan12, Ing3* and *Cped1* located on Chromosome six are known to promote proliferation of breast cancer cells (Yang et al., 2023), be a critical factor for cancer associated fibroblast mediated invasion (Otomo et al., 2014), used as a potential biomarker for CRC/breast cancer (Kim and Lee, 2022; Li et al., 2023) or confer oncogenic effects in prostate cancer (Zhu et al., 2023) respectively. Likewise on chromosome 12, expression of *Matn3, Osr1, and Nt5c1b* have been associated with colon adenocarcinoma, gastric and breast cancer development (Zhao Z. et al., 2023a; Chi et al., 2023; Gadwal et al., 2023); potential use as a biomarker for breast cancer (Li et al., 2020) or promotion of EMT and metastasis in breast cancer (Wang et al., 2020; Li et al., 2021) and identified as a cancer testis-antigen in canine malignancies (Nemec et al., 2019) respectively. On the X chromosome, *Il13ra1, Pgrmc1, Septin6, Ndufa1, Nkrf, Zcchc12, Lonrf3, Slc25a5, Ct47 and Dock11* have been associated with numerous cancers including: breast cancer (Park et al., 2017; Zhao Y. et al., 2023), basal cell carcinoma (Mamelak et al., 2005), glioblastoma multiforme (Han and Puri, 2018), gastric cancer (Li et al., 2019), hepatocellular carcinoma (Fan et al., 2021), lung cancer (Lu et al., 2023), neuroblastoma (Seneviratne et al., 2023), osteosarcoma (Cui and Dong, 2022), ovarian serous cystadenocarcinoma (Zhang et al., 2018), pancreatic cancer (Feng et al., 2021; Shi et al., 2022), papillary thyroid cancer (Wang et al., 2017) and T-cell acute lymphoblastic leukemia (Lahera et al., 2023). Whereas members of the A kinase anchor (AKAP) and Reproductive homeobox (Rhox) protein families are associated with various cancers (MacLean, 2013; Reggi and Diviani, 2017; Vaughan-Shaw et al., 2021). Interestingly, a recent study has identified a deletion mutant of *Slc25a5* associated with familial predisposition to mesothelioma (Akarsu et al., 2023). In contrast to other CC based studies, our analysis did not identify any specific CCMT candidate modifier genes at any of the major effect QTLs that had a significant influence on the phenotype, but rather our data suggests a complex polygenic interplay in which small variations in multiple host genes affect ARD survival/latency.

To gain a better understanding of whether CCMT candidate modifier genes influenced human mesothelioma, we assessed how the expression of human homologues of CCMT candidate modifier genes affected survival outcome in two independent human mesothelioma transcriptomic datasets. These cohorts had different data that could be used as surrogates for disease latency: age at surgery (Bueno cohort) or age at diagnosis (TCGA-MESO cohort). Using either of these to estimate disease latency, we identified eight human homologues of the candidate CCMT modifier genes that significantly influenced survival outcome. Expression of *CPED1* or *OSR1* was associated with a poorer survival outcome in the Bueno cohort. These data are consistent with other studies in which a novel Cadherin-like and PC-esterase domain containing 1 (CPED1) and Forkhead box protein P2 (FOXP2) fusion product (FOXP2-CPED1) was shown to confer oncogenic effects in prostate cancer (Zhu et al., 2023) and overexpression of *CPED1* touted as a potential prognostic signature in stomach adenocarcinoma (Zhou et al., 2020). Similarly, high expression of Odd-skipped related transcription factor 1 (OSR1) has been used as a predictive biomarker for poor prognosis and linked to lymph node metastases in breast cancer (Li et al., 2020; Li et al., 2021). Conversely, *OSR1* has also been identified as a tumour suppressor gene in breast cancer, where reduced expression promotes breast cancer proliferation and invasion (Wang et al., 2020; Yu and Ouyang, 2022).

In the TCGA-Meso cohort, higher expression of *HS1BP3*, *IL13RA1*, *LSM8*, *NDUFA1* or *TES* was associated with a significantly poorer survival outcome. Only expression of *TSPAN12* was associated with improved outcome. Tetraspanin-12 plays a critical role in cancer fibroblast cell mediant-contact inhibition (Otomo et al., 2014) and consistent with our study, *TSPAN12* expression is associated with a favourable survival outcome in ovarian cancer (Ji et al., 2019). Furthermore, elevated expression of *IL13RA1* has also been associated with poor prognosis in patients with invasive breast cancer (Park et al., 2017), poor prognosis and drug resistance in glioblastoma multiforme patients (Han and Puri, 2018) and overexpression of LSM2-(8) is associated with poor prognosis in cutaneous melanoma skin cancer (Liu et al., 2023); outcomes consistent with expression profiles observed in the TCGA-MESO cohort. We note that the increased expression of *NDUFA1*, *TES* and *IL13RA1* associated with poor survival outcome in the TCGA mesothelioma cohort contrasted with the literature. Downregulation or loss of *NDUFA1* expression is a known consistent feature of basal cell carcinoma (Mamelak et al., 2005), while reduced expression of *IL13RA1* has been associated with apoptosis and promotion of epithelial to mesenchymal transition (EMT) in pancreatic cancer (Shi et al., 2022). Like *OSR1*, *TES* (testin LIM domain protein) functions as a Mena-dependent tumour suppressor gene in many cancers, including gastric cancer (Wang et al., 2019), with loss of expression associated with cancer progression. However, *TES* overexpression has been noted in progressive cervical intraepithelial neoplasia (Popiel et al., 2021).

Interestingly, we did not identify any homologues of CCMT candidate modifier genes that were common to both mesothelioma cohorts, as the genes identified as significant in the Bueno cohort were not identified as significant in the TCGA-MESO cohort and visa-versa. However, this is not surprising given that the major effect QTL affecting overall CCMT survival was derived from ARD latency (i.e., the time from first asbestos exposure to first signs of disease), and not once ARD was established (i.e., disease progression); which is essentially what both human mesothelioma datasets represent. While we acknowledge this as a limitation of this study, we also recognise that few, if any publicly available mesothelioma datasets exist that incorporate sufficient “disease latency” data, and these are limited to smaller studies on familial predisposition to mesothelioma (Akarsu et al., 2023).

In the last decade, we have made significant advances in our understanding of the genomics of mesothelioma. We have gained detailed insight into end stage disease; identifying the genetic mutations that characterise mesothelioma; understanding the biological pathways involved in mesothelioma development helping to aid the development of novel therapeutic options. However, despite much effort, our understanding of how host genetics influences mesothelioma development and the discovery of a common set of mesothelioma-specific host risk-alleles remains elusive.

In conclusion, this is the first study to apply the power of the Collaborative Cross murine model to identify host genetic factors that influence mesothelioma development. Using this strategy, we demonstrated that host genetics does impact ARD development. Importantly, the effect of host genetics was not observed after ARD disease was established, demonstrating that host genetics is unable to slow disease progression in this model once the tumour is established. We further identified three major effect QTL, across multiple chromosomes, which involved numerous genes with known cancer associations, including many that have not previously been linked to mesothelioma or asbestos related disease development, validating the feasibility of our gene discovery approach. Additionally, eight human homologues of CCMT candidate modifier genes were identified as having a significant influence in survival outcome in two independent human mesothelioma datasets. Our study confirms the feasibility of this novel approach and provides a rational framework required for identification of multiple low risk gene variants that has so far eluded conventional human mesothelioma genetic studies.

## Data Availability

The datasets presented in this study can be found in online repositories. The names of the repository/repositories and accession number(s) can be found in the article/Supplementary Material.

## Supplementary material

SUPPLEMENTARY FIGURE S1
**(A)** Genome-wide scan based on median ascites volume from 71 asbestos exposed CCMT strains depicting highly suggestive QTL on chromosomes 2, 3, 4, 5, 9, 16, and X (red arrows). Genotyping, construction of CC strain haplotypes, and linkage analysis were performed as previously described (Ram et al., 2014). Phenotype data was analysed using the GeneMiner MugaQTL(as is) setting. The x-axis shows the chromosomal position, and the y-axis shows the 2log10(P) values; the *p*-values were derived from the linkage haplotype data. **(B)** Top: Plot of LOD scores along chromosomes 2, 3, 4, 5, 9, 16, and X. Bottom: Founder allele coefficients: plot of the calculated log-odds ratio of eight founder alleles over the chromosome where the founders are color-coded. Dotted line highlights chromosomal region containing peak QTL.

SUPPLEMENTARY TABLE S1 CCMT and MexTAg control group characteristics. ARD, asbestos related disease; Asb, asbestos; M, male; F, female.

SUPPLEMENTARY TABLE S2 CCMT Phenotype data. ARD, asbestos related disease; Vol, volume; mL, milliliter; SD, standard deviation.

SUPPLEMENTARY TABLE S3 Univariate analysis results for histology disease progression using general linear regression.

SUPPLEMENTARY TABLE S4 Genes located within ±10 Mb of peak QTL associated with median CCMT survival / latency. Bold genes occur at or span peak QTL. Highlighted genes identified as significantly impacting outcome in Bueno (green) and TCGA (orange) human mesothelioma datasets. [74], [75], [76], [77], [78].

SUPPLEMENTARY TABLE S5 Genes located within ±10 Mb of peak QTL associated with median ascites volume (mL).

SUPPLEMENTARY TABLE S6 Univariate cox-regression analysis using the using the “coxph” function from the “survival” R package of 211 human mesothelioma patients in the Bueno dataset using age at the time of surgery as a surrogate for survival. Only CCMT candidate genes with known human gene homologues were included in the analysis. Genes in bold (green highlight) had significant association with outcome. HR, Hazard ratio.

SUPPLEMENTARY TABLE S7 Univariate cox-regression analysis using the using the “coxph” function from the “survival” R package of 74 human mesothelioma patients in the TCGA dataset using age at the time of diagnosis as a surrogate for survival. Only CCMT candidate genes with known human gene homologues were included in the analysis. Genes in bold (orange highlight) had significant association with outcome. HR, Hazard ratio.
