# Supplementary material for: The MexTAg collaborative cross: host genetics affects asbestos related disease latency, but has little influence once tumours develop
Source: Front Toxicol. 2024 Apr 17;6:1373003. doi: 10.3389/ftox.2024.1373003 (PMC11061428; doi:10.3389/ftox.2024.1373003)
Supplement: Supplementary file 1 [file Table4.pdf]

Supplemental Table S4: Genes located within ±10Mb of peak QTL associated with median CCMT survival / latency. **Bold** genes occur at or span peak QTL. Highlighted genes identified as significantly influencing outcome in Bueno (green) and TCGA (orange) human mesothelioma datasets. BC, breast cancer; BCC, basal cell carcinoma; Ca-T-lymp*, canine T-cell lymphoma; CIN, cervical intraepithelial neoplasia; COAD, colon adenocarcinoma; CRC, colorectal cancer; GBM, glialblastoma multiforme; GC, gastric cancer; HCC, hepatocellular carcinoma; LC, lung cancer; LUAD, lung adenocarcinoma; Meso, mesothelioma; NBL, neuroblastoma; NSCLC, non-small cell lung cancer; OC, ovarian cancer; OS, osteosarcoma; OSC, ovarian serous cystadenocarcinoma; OXPHOS, oxidative phosphorylation; Panc, pancreatic cancer; PC, prostate cancer; PTC, papillary thyroid cancer; RC, renal cancer; SKCM, cutaneous melanoma skin cancer; STAD, stomach adenocarcinoma; T-ALL, T-cell acute lymphoblastic leukemia; TNBC, triple negative breast cancer.

Chromosome	*Gene ID*	*Gene Description*	*Cancer Association*	*Citations*
6	*Tes*	testin LIM domain protein	GC, CIN	[73; 74]
	Cav2	caveolin 2		
	Cav1	caveolin 1, caveolae protein		
	Met	met proto-oncogene		
	Capza2	capping actin protein of muscle Z-line subunit alpha 2		
	St7	suppression of tumorigenicity 7		
	Wnt2	wingless-type MMTV integration site family, member 2		
	Asz1	ankyrin repeat, SAM and basic leucine zipper domain containing 1		
	Cftr	cystic fibrosis transmembrane conductance regulator		
	Cttnbp2	cortactin binding protein 2		
	Lsm8	LSM8 homolog, U6 small nuclear RNA associated	GC, SKCM	[72]
	Ankrd7	ankyrin repeat domain 7		
	Kcnd2	**potassium voltage-gated channel, Shal-related family, member 2**	BC, GC, LUAD	[39; 75; 76]
	Tspan12	**tetraspanin 12**	NSCLC, HCC, OC	[40; 71]
	Ing3	**inhibitor of growth family, member 3**	BC, CRC	[41; 42]
	Cped1	**cadherin-like and PC-esterase domain containing 1**	PC, STAD	[43; 69]
	Wnt16	wingless-type MMTV integration site family, member 16		
	Fam3c	FAM3 metabolism regulating signaling molecule C		
	Ptprz1	protein tyrosine phosphatase, receptor type Z, polypeptide 1		
12	*Apob*	apolipoprotein B		
	Ldah	lipid droplet associated hydrolase		
	Gdf7	growth differentiation factor 7		
	Hs1bp3	HCLS1 binding protein 3	HCC	[77]
	Rhob	ras homolog family member B		
	Slc7a15	solute carrier family 7 (cationic amino acid transporter, y+ system), member 15		
	Pum2	pumilio RNA-binding family member 2		
	Sdc1	syndecan 1		
	Laptm4a	lysosomal-associated protein transmembrane 4A		
	Matn3	**matrilin 3**	BC, GC, COAD	[44; 45; 46]
	Wdr35	**WD repeat domain 35**		
	Ttc32	**tetratricopeptide repeat domain 32**	RC, LC	

	Osr1	**odd-skipped related transcription factor 1**	BC, OC	[47; 48; 49; 70]
	Nt5c1b	**5'-nucleotidase, cytosolic IB**	Ca-T-lymp*	[50]
	Rdh14	**retinol dehydrogenase 14 (all-trans and 9-cis)**		
	Kcns3	potassium voltage-gated channel, delayed-rectifier, subfamily S, member 3		
	Msgn1	mesogenin 1		
	Gen1	GEN1, Holliday junction 5' flap endonuclease		
	Smc6	structural maintenance of chromosomes 6		
	Vsnl1	visinin-like 1		
	Rad51ap2	RAD51 associated protein 2		
	Cyria	CYFIP related Rac1 interactor A		
	Mycn	v-myc avian myelocytomatosis viral related oncogene, neuroblastoma derived		
	Ddx1	DEAD box helicase 1		
	Nbas	neuroblastoma amplified sequence		
X	*C1galt1c1*	C1GALT1-specific chaperone 1		
	Nkap	NFKB activating protein		
	Rpl39	ribosomal protein L39		
	Atp1b4	ATPase Na+/K+ transporting, beta 4 polypeptide		
	Upf3b	UPF3 regulator of nonsense transcripts homolog B (yeast)		
	Mcts1	malignant T cell amplified sequence 1		
	Rnf113a1	ring finger protein 113A1		
	Ube2a	ubiquitin-conjugating enzyme E2A		
	Il13ra1	**interleukin 13 receptor, alpha 1**	Panc, GBM, BC	[51; 54; 62]
	Lamp2	lysosomal-associated membrane protein 2		
	Pgrmc1	**progesterone receptor membrane component 1**	TNBC	[52]
	Septin6	**septin 6**	HCC, T-ALL	[56; 64]
	Rhox9	reproductive homeobox 9		
	Zbtb33	zinc finger and BTB domain containing 33		
	Ndufa1	**NADH:ubiquinone oxidoreductase subunit A1**	OXPHOS, BCC	[53; 78]
	Nkrf	**NF-kappaB repressing factor**	LC	[57]
	Steep1	**STING1 ER exit protein 1**		
	6030498E09Rik	RIKEN cDNA 6030498E09 gene		
	Rhox4b	reproductive homeobox 4B		
	Akap17b	**A kinase anchor protein 17B**	many	[66]
	Cul4b	cullin 4B		
	Zcchc12	**zinc finger, CCHC domain containing 12**	OS, PTC	[59; 63]
	Rhox13	reproductive homeobox 13		
	Lonrf3	**LON peptidase N-terminal domain and ring finger 3**	Panc	[61]
	Rhox2a	reproductive homeobox 2A		
	Slc25a5	**solute carrier family 25 (mitochondrial carrier, adenine nucleotide translocator), member 5**	Meso, GC, NBL	[55; 58; 68]
	Ct47	**cancer/testis antigen 47**	many	[79]
	Rhox2g	reproductive homeobox 2G		
	Rhox2d	reproductive homeobox 2D		
	Gm14819	**predicted gene 14819**		
	Rhox8	reproductive homeobox 8		

	Rhox1	**reproductive homeobox 1**	many	[65]
	Rhox4a	reproductive homeobox 4A		
	Rhox7a	reproductive homeobox 7A		
	Rhox10	reproductive homeobox 10		
	Btg1b	BTG anti-proliferation factor 1B		
	Btg1c	BTG anti-proliferation factor 1C		
	Rhox4c	reproductive homeobox 4C		
	Rhox4d	reproductive homeobox 4D		
	Rhox4e	reproductive homeobox 4E		
	Rhox4f	reproductive homeobox 4F		
	Rhox4g	reproductive homeobox 4G		
	Rhox3h	reproductive homeobox 3H		
	Rhox7b	reproductive homeobox 7B		
	Gm14569	**predicted gene 14569**		
	Gm10486	**predicted gene 10486**		
	Rhox2h	reproductive homeobox 2H		
	Akap14	**A kinase anchor protein 14**	CRC	[67]
	Dock11	**dedicator of cytokinesis 11**	OSC	[60]
	Rhox3a	reproductive homeobox 3A		
	Sowahd	sosondowah ankyrin repeat domain family member D		
	Tmem255a	transmembrane protein 255A		
	Rhox6	reproductive homeobox 6		
	Rhox11	reproductive homeobox 11		
	Slc25a43	solute carrier family 25, member 43		
	Gm9	predicted gene 9		
	Rhox12	reproductive homeobox 12		
	Rhox2b	reproductive homeobox 2B		
	Rhox2c	reproductive homeobox 2C		
	Rhox3c	reproductive homeobox 3C		
	Rhox2e	reproductive homeobox 2E		
	Rhox2f	reproductive homeobox 2F		
	Rhox3f	reproductive homeobox 3F		
	Rhox3e	reproductive homeobox 3E		
	Rhox3g	reproductive homeobox 3G		
