# Supplementary material for: The MexTAg collaborative cross: host genetics affects asbestos related disease latency, but has little influence once tumours develop
Source: Front Toxicol. 2024 Apr 17;6:1373003. doi: 10.3389/ftox.2024.1373003 (PMC11061428; doi:10.3389/ftox.2024.1373003)
Supplement: Supplementary file 2 [file Table1.docx]

**Supplemental Table S1**: **CCMT and MexTAg control group characteristics.**

ARD = asbestos related disease; Asb = asbestos; M = male; F= female

| **Parental**  **Strain (72)** | **CC-MexTAg**  **strain** | **No#**  **Mice** | **Sex** | | **Median age @**  **first Asb exposure (wks)** | | **Non-ARD death** | **ARD death** | **ARD**  **Incidence** |
| --- | --- | --- | --- | --- | --- | --- | --- | --- | --- |
|  |  |  | **M** | **F** | **Age** | **Range** |  |  |  |
| ***Cohort total*** | ***72*** | ***2565*** | ***1285*** | ***1280*** | ***11.1*** | ***5.7-38.6*** | ***333*** | ***2232*** | ***87%*** |
| 266-MexTAg Het | 266-MexTAg Het | 32 | 13 | 19 | 10.6 | 6.4 – 15 | 2 | 30 | 93.8% |
| BEM | 266-BEM_AG | 38 | 16 | 22 | 12.4 | 6.4 – 23 | 1 | 37 | 97.4% |
| BOM | 266-BOM_GB | 41 | 24 | 17 | 7.6 | 6 – 19.7 | 7 | 34 | 82.9% |
| BOON | 266-BOON_HF | 35 | 14 | 21 | 10.0 | 6.7 – 13.4 | 7 | 28 | 80.0% |
| CAMERON | 266-CAMERON_GA | 25 | 13 | 12 | 20.4 | 6 – 28.9 | 0 | 25 | 100.0% |
| CC001 | 266-CC001/Unc | 32 | 15 | 17 | 15.2 | 6.6 – 17 | 6 | 26 | 81.3% |
| CC017 | 266-CC017/Unc | 32 | 13 | 19 | 9.4 | 6.1 – 14 | 5 | 27 | 84.4% |
| CC021 | 266-CC021/Unc | 29 | 12 | 17 | 7.7 | 7.3 – 8.3 | 2 | 27 | 93.1% |
| CC035 | 266-CC035/Unc | 29 | 13 | 16 | 12.1 | 6.4 -12.6 | 4 | 25 | 86.2% |
| CC036 | 266-CC036/Unc | 27 | 12 | 15 | 11.6 | 6.4 – 13 | 0 | 27 | 100.0% |
| CC040 | 266-CC040/TauUnc | 33 | 15 | 18 | 6.6 | 5.9 – 6.9 | 1 | 32 | 97.0% |
| CC041 | 266-CC041/TauUnc | 31 | 16 | 15 | 9.9 | 6.3 -16.1 | 0 | 31 | 100.0% |
| CC044 | 266-CC044/Unc | 40 | 18 | 22 | 8.9 | 6.1 – 15.1 | 7 | 33 | 82.5% |
| CC046 | 266-CC046/Unc | 40 | 22 | 18 | 13.1 | 6 – 14.7 | 9 | 31 | 77.5% |
| CC053 | 266-CC053/Unc | 36 | 21 | 15 | 8.3 | 6.4 – 12.6 | 6 | 30 | 83.3% |
| CC057 | 266-CC057/Unc | 35 | 20 | 15 | 14.9 | 6.1 – 16.6 | 5 | 30 | 85.7% |
| CC058 | 266-CC058/Unc | 35 | 15 | 20 | 8.0 | 6.3 – 14.7 | 2 | 33 | 94.3% |
| CC059 | 266-CC059/TauUnc | 32 | 20 | 12 | 8.3 | 7 – 14.4 | 2 | 30 | 93.8% |
| CC060 | 266-CC060/Unc | 31 | 17 | 14 | 10.4 | 7.1 – 14.6 | 4 | 27 | 87.1% |
| CC074 | 266-CC074/Unc | 45 | 23 | 22 | 8.1 | 7.4 – 15 | 5 | 40 | 88.9% |
| CC081 | 266-CC081/Unc | 25 | 11 | 14 | 20.5 | 5.9 – 23.6 | 4 | 21 | 84.0% |
| CIS | 266-CIS_AD | 35 | 19 | 16 | 14.1 | 6.6 – 20.9 | 3 | 32 | 91.4% |
| DAVIS | 266-DAVIS_BA | 29 | 16 | 13 | 13.0 | 6.3 – 18.4 | 4 | 25 | 86.2% |
| DONNELL | 266-DONNELL_HA | 39 | 21 | 18 | 11.7 | 7 – 18.4 | 1 | 38 | 97.4% |
| FEW | 266-FEW_FD | 37 | 27 | 10 | 8.1 | 6.1 – 10.7 | 8 | 29 | 78.4% |
| FIM | 266FIM-DF | 38 | 15 | 23 | 9.9 | 7.1 12.6 | 5 | 33 | 86.8% |
| FIV | 266-FIV-AC | 42 | 20 | 22 | 9.7 | 7 – 11.7 | 2 | 40 | 95.2% |
| FUF | 266-FUF_HE | 31 | 18 | 13 | 13.6 | 7.6 – 19.7 | 4 | 27 | 87.1% |
| GIG | 266-GIG_EF | 43 | 20 | 23 | 11.1 | 7.3 – 13.9 | 11 | 32 | 74.4% |
| GIT | 266-GIT_GC | 40 | 22 | 18 | 11.3 | 6.6 – 16.1 | 3 | 37 | 92.5% |
| HAX2 | 266-HAX2_EF | 40 | 17 | 23 | 10.3 | 6.1 – 12 | 6 | 34 | 85.0% |
| HAZ | 266-HAZ_FE | 40 | 26 | 14 | 8.7 | 6 – 131 | 4 | 36 | 90.0% |
| HIP | 266-HIP_GA | 31 | 19 | 12 | 19.6 | 5.9 – 29.1 | 3 | 28 | 90.3% |
| JUD | 266-JUD_EF | 32 | 16 | 16 | 9.1 | 6.3 – 11.3 | 1 | 31 | 96.9% |
| JUNIOR | 266-JUNIOR_GB | 39 | 20 | 19 | 10.0 | 7 – 11 | 6 | 33 | 84.6% |
| KAV | 266-KAV_AF | 28 | 11 | 17 | 6.9 | 6.4 – 18.9 | 1 | 27 | 96.4% |
| LAM | 266-LAM_HD | 37 | 17 | 20 | 15.1 | 7.9 – 16.9 | 7 | 30 | 81.1% |
| LAT | 266-LAT_AC | 31 | 14 | 17 | 6.4 | 6.3 – 12.9 | 2 | 29 | 93.5% |
| LAX | 266-LAX_FC | 40 | 23 | 17 | 14.1 | 6.6 – 19.4 | 7 | 33 | 82.5% |
| LEM | 266-LEM_AF | 38 | 17 | 21 | 11.4 | 6.1 – 14 | 4 | 34 | 89.5% |
| LEM2 | 266-LEM2_AF | 42 | 21 | 21 | 12.1 | 5.7 – 15.7 | 7 | 35 | 83.3% |
| LIL | 266-LIL_AF | 40 | 15 | 25 | 8.0 | 6 – 14.1 | 5 | 35 | 87.5% |
| LIV | 266-LIV_DA | 32 | 16 | 16 | 10.6 | 6.3 – 14.3 | 12 | 20 | *62.5%* |
| LOD | 266-LOD_AE | 35 | 15 | 20 | 8.3 | 6.1 – 12.1 | 9 | 26 | 74.3% |
| LOT | 266-LOT_FC | 40 | 22 | 18 | 8.3 | 5.3 – 10.4 | 5 | 35 | 87.5% |
| LOX | 266-LOX_GF | 34 | 15 | 19 | 11.0 | 5.9 – 18.9 | 3 | 31 | 91.2% |
| LUF | 266-LUF_AD | 40 | 18 | 22 | 11.7 | 6.3 – 13.4 | 13 | 27 | *67.5%* |
| LUS | 266-LUS_AH | 30 | 14 | 16 | 6.9 | 6.7 – 10.1 | 1 | 29 | 96.7% |
| LUV | 266-LUV_DG | 41 | 21 | 20 | 10.6 | 6.3 – 15.7 | 2 | 39 | 95.1% |
| MEE | 266-MEE_AG | 38 | 15 | 23 | 13.6 | 7.1 – 17 | 7 | 31 | 81.6% |
| NUK | 266-NUK_AC | 35 | 19 | 16 | 8.3 | 5.7 – 17.4 | 5 | 30 | 85.7% |
| PEF | 266-PEF_EC | 32 | 16 | 16 | 9.4 | 9.1 – 10 | 2 | 30 | 93.8% |
| PEF2 | 266-PEF2_EC | 30 | 20 | 10 | 9.4 | 5.7 -12.1 | 0 | 30 | 100.0% |
| PIPING | 266-PIPING_BD | 40 | 18 | 22 | 11.6 | 6 – 26.9 | 5 | 35 | 87.5% |
| POH | 266-POH_DC | 30 | 16 | 14 | 7.9 | 6.1 – 14 | 2 | 28 | 93.3% |
| PUB | 266-PUB_CD | 43 | 23 | 20 | 10.3 | 6.9 – 13.1 | 2 | 41 | 95.3% |
| ROGAN | 266-ROGAN_CF | 44 | 26 | 18 | 12.3 | 6.6 – 14.1 | 8 | 36 | 81.8% |
| SAT | 266-SAT_GA | 40 | 20 | 20 | 9.6 | 7.3 – 12 | 1 | 39 | 97.5% |
| SHE | 266-SHE_AH | 38 | 12 | 26 | 12.3 | 6 – 13.6 | 19 | 19 | *50.0%* |
| TAS | 266-TAS_FE | 40 | 19 | 21 | 14.7 | 6 - 17 | 3 | 37 | 92.5% |
| TOFU | 266-TOFU_FB | 39 | 18 | 21 | 16.0 | 6.9 - 22.3 | 5 | 34 | 87.2% |
| TOP | 266-TOP_DA | 38 | 26 | 12 | 13.0 | 6.1 - 15 | 5 | 33 | 86.8% |
| VIT | 266-VIT_ED | 30 | 17 | 13 | 23.9 | 6 - 29 | 3 | 27 | 90.0% |
| VUX2 | 266-VUX2_HF | 35 | 23 | 12 | 25.3 | 6.1 - 29.4 | 1 | 34 | 97.1% |
| WAB2 | 266-WAB2_DH | 41 | 19 | 22 | 13.3 | 7 - 25 | 11 | 30 | 73.2% |
| WAD | 266-WAD_HG | 18 | 14 | 4 | 19.5 | 19 - 20 | 1 | 17 | 94.4% |
| WOB2 | 266-WOB2_DH | 43 | 24 | 19 | 15.3 | 8.1 - 18.9 | 5 | 38 | 88.4% |
| XAC2 | 266-XAC2_HG | 25 | 13 | 12 | 35.4 | 6.4 - 38.6 | 5 | 20 | 80.0% |
| XAK | 266-XAK_AG | 44 | 20 | 24 | 9.6 | 6.1 - 12.7 | 4 | 40 | 90.9% |
| YID | 266-YID_FH | 43 | 14 | 29 | 15.1 | 8 - 25 | 6 | 37 | 86.0% |
| ZIE2 | 266-ZIE2_AH | 39 | 18 | 21 | 11.9 | 6.9 - 17 | 13 | 26 | *66.7%* |
| ZIF2 | 266-ZIF2_FC | 33 | 17 | 16 | 18.6 | 7 - 22.1 | 2 | 31 | 93.9% |

Grey highlight >2 standard deviations from cohort mean
