# Supplementary material for: The MexTAg collaborative cross: host genetics affects asbestos related disease latency, but has little influence once tumours develop
Source: Front Toxicol. 2024 Apr 17;6:1373003. doi: 10.3389/ftox.2024.1373003 (PMC11061428; doi:10.3389/ftox.2024.1373003)
Supplement: Supplementary file 3 [file Table7.docx]

**Supplemental Table S7: Univariate cox-regression analysis using the using the ‘coxph’ function from the ‘survival’ R package of 74 human mesothelioma patients in the TCGA dataset using age at the time of diagnosis as a surrogate for survival.** Only CCMT candidate genes with known human gene homologues were included in the analysis. Genes in **bold** (orange highlight) had significant association with outcome. HR = Hazard ratio.

| **Covariates** | **beta** | **HR (95% CI for HR)** | **wald.test** | **p.value** |
| --- | --- | --- | --- | --- |
| Sex | -0.0033 | 1 (0.5-2) | 0 | 0.99 |
| *WNT16* | -0.0051 | 0.99 (0.6-1.7) | 0 | 0.98 |
| *ST7* | 0.15 | 1.2 (0.7-1.9) | 0.34 | 0.56 |
| *SLC25A5* | 0.071 | 1.1 (0.64-1.8) | 0.07 | 0.79 |
| *STEEP1* | -0.035 | 0.97 (0.58-1.6) | 0.02 | 0.89 |
| *LAPTM4A* | 0.35 | 1.4 (0.85-2.4) | 1.8 | 0.19 |
| *ING3* | 0.14 | 1.2 (0.69-1.9) | 0.29 | 0.59 |
| *CTTNBP2* | 0.33 | 1.4 (0.81-2.4) | 1.4 | 0.23 |
| *SLC25A43* | 0.068 | 1.1 (0.64-1.8) | 0.07 | 0.79 |
| *DDX1* | 0.11 | 1.1 (0.67-1.9) | 0.19 | 0.66 |
| *APOB* | -0.19 | 0.83 (0.49-1.4) | 0.51 | 0.48 |
| *PGRMC1* | -0.092 | 0.91 (0.55-1.5) | 0.13 | 0.72 |
| *NKAP* | 0.24 | 1.3 (0.77-2.1) | 0.87 | 0.35 |
| *CAV2* | 0.21 | 1.2 (0.74-2.1) | 0.65 | 0.42 |
| *CAV1* | -0.2 | 0.82 (0.48-1.4) | 0.57 | 0.45 |
| *MET* | 0.38 | 1.5 (0.86-2.5) | 2 | 0.16 |
| *WNT2* | 0.22 | 1.2 (0.75-2.1) | 0.72 | 0.4 |
| ***TSPAN12*** | **-0.59** | **0.55 (0.32-0.95)** | **4.7** | **0.03** |
| *CPED1* | -0.32 | 0.73 (0.44-1.2) | 1.5 | 0.22 |
| *PTPRZ1* | 0.11 | 1.1 (0.67-1.9) | 0.18 | 0.67 |
| *SDC1* | -0.023 | 0.98 (0.58-1.7) | 0.01 | 0.93 |
| ***HS1BP3*** | **0.67** | **2 (1.1-3.4)** | **5.8** | **0.016** |
| *LDAH* | 0.18 | 1.2 (0.71-2) | 0.46 | 0.5 |
| *WDR35* | -0.12 | 0.89 (0.53-1.5) | 0.21 | 0.65 |
| *RNF113A* | 0.11 | 1.1 (0.67-1.9) | 0.17 | 0.68 |
| *SEPTIN6* | 0.25 | 1.3 (0.77-2.2) | 0.94 | 0.33 |
| *TMEM255A* | -0.097 | 0.91 (0.55-1.5) | 0.14 | 0.71 |
| ***NDUFA1*** | **0.56** | **1.8 (1-3)** | **4.4** | **0.035** |
| ***LSM8*** | **0.63** | **1.9 (1.1-3.2)** | **5.3** | **0.021** |
| ***IL13RA1*** | **0.74** | **2.1 (1.2-3.5)** | **7.6** | **0.0057** |
| *MATN3* | 0.071 | 1.1 (0.64-1.8) | 0.07 | 0.79 |
| ***TES*** | **0.73** | **2.1 (1.2-3.6)** | **7.2** | **0.0075** |
| *SYCP3* | 0.2 | 1.2 (0.73-2.1) | 0.57 | 0.45 |
| *OSR1* | -0.17 | 0.84 (0.51-1.4) | 0.42 | 0.52 |
| *GDF7* | -0.074 | 0.93 (0.56-1.5) | 0.08 | 0.78 |
| *RHOB* | -0.24 | 0.79 (0.47-1.3) | 0.85 | 0.36 |
| *SMC6* | 0.14 | 1.2 (0.69-1.9) | 0.29 | 0.59 |
| *VSNL1* | 0.23 | 1.3 (0.75-2.1) | 0.75 | 0.39 |
| *ZCCHC18* | -0.046 | 0.96 (0.57-1.6) | 0.03 | 0.86 |
| *KCNS3* | 0.16 | 1.2 (0.7-2) | 0.37 | 0.54 |
| *C1GALT1C1* | -0.21 | 0.81 (0.49-1.4) | 0.62 | 0.43 |
| *ZCCHC12* | 0.29 | 1.3 (0.8-2.2) | 1.3 | 0.26 |
| *LONRF3* | 0.086 | 1.1 (0.65-1.8) | 0.11 | 0.74 |
| *ZBTB33* | -0.074 | 0.93 (0.56-1.6) | 0.08 | 0.78 |
| *GEN1* | 0.053 | 1.1 (0.63-1.8) | 0.04 | 0.84 |
| *TTC32* | -0.21 | 0.81 (0.48-1.4) | 0.6 | 0.44 |
| *KCND2* | -0.044 | 0.96 (0.58-1.6) | 0.03 | 0.87 |
| *SOWAHD* | 0.31 | 1.4 (0.81-2.3) | 1.3 | 0.25 |
| *FAM3C* | 0.16 | 1.2 (0.7-1.9) | 0.36 | 0.55 |
| *CYRIA* | 0.26 | 1.3 (0.77-2.2) | 0.95 | 0.33 |
| *RPL39* | 0.017 | 1 (0.61-1.7) | 0 | 0.95 |
| *MCTS1* | 0.27 | 1.3 (0.79-2.2) | 1.1 | 0.3 |
| *RDH14* | -0.028 | 0.97 (0.58-1.6) | 0.01 | 0.91 |
