# Supplementary figures and images for: The MexTAg collaborative cross: host genetics affects asbestos related disease latency, but has little influence once tumours develop
Source: Front Toxicol. 2024 Apr 17;6:1373003. doi: 10.3389/ftox.2024.1373003 (PMC11061428; doi:10.3389/ftox.2024.1373003)

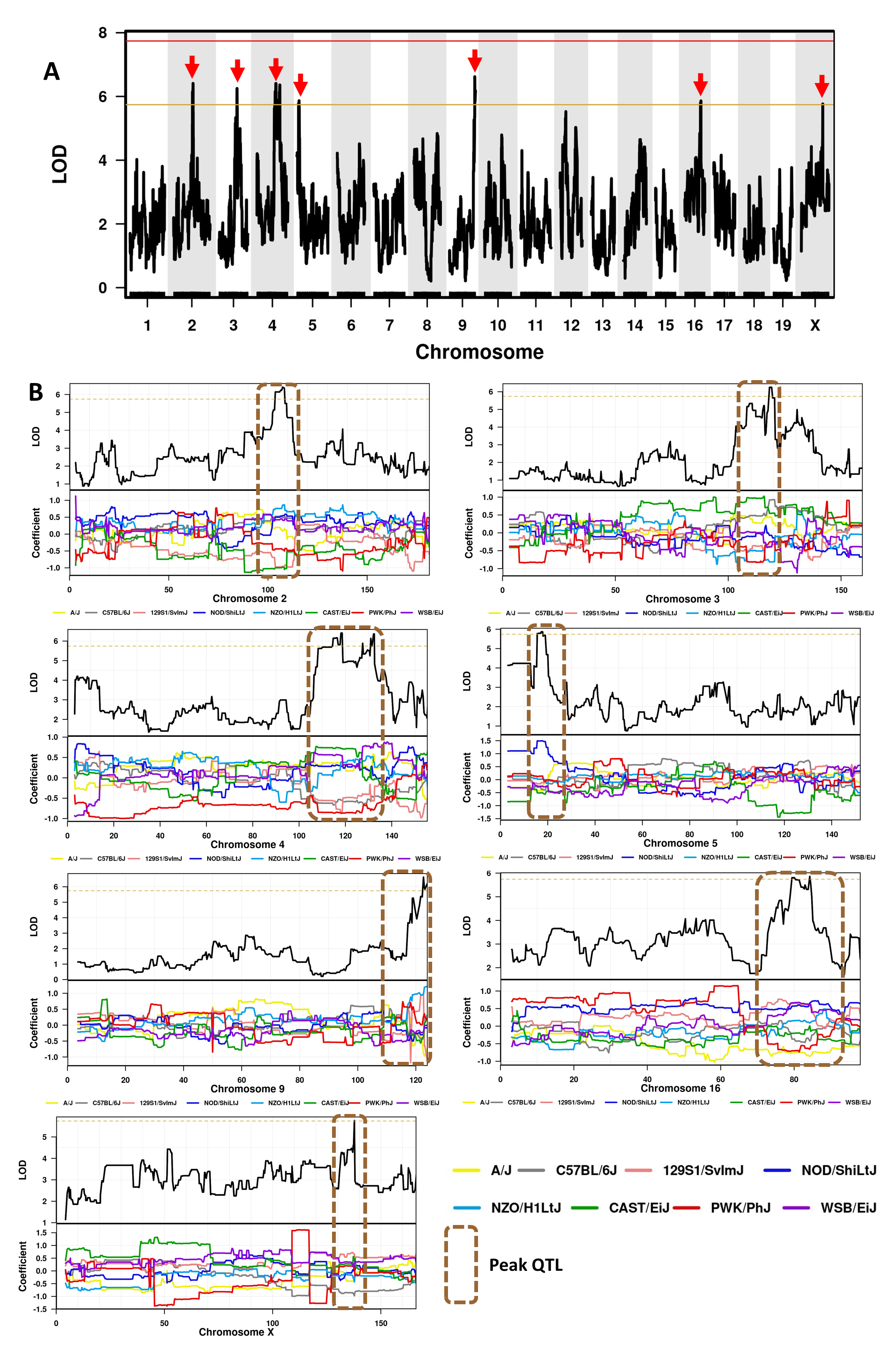

Supplement: Supplementary file 4 [file Image1.tif]
