# Supplementary material for: The MexTAg collaborative cross: host genetics affects asbestos related disease latency, but has little influence once tumours develop
Source: Front Toxicol. 2024 Apr 17;6:1373003. doi: 10.3389/ftox.2024.1373003 (PMC11061428; doi:10.3389/ftox.2024.1373003)
Supplement: Supplementary file 5 [file Table2.docx]

**Supplemental Table S2**: **CCMT Phenotype data.**

ARD = asbestos related disease; Vol = volume; ml =milliliter, SD = standard deviation

| **CC-MexTAg strain** | **Overall Survival (days)** | | | | **ARD Latency (days)** | | | | **ARD Progression (days)** | | | | **Ascites Vol (ml)** | | | |
| --- | --- | --- | --- | --- | --- | --- | --- | --- | --- | --- | --- | --- | --- | --- | --- | --- |
|  | **Median** | **Min** | **Max** | **SD** | **Median** | **Min** | **Max** | **SD** | **Median** | **Min** | **Max** | **SD** | **Median** | **Min** | **Max** | **SD** |
| ***Cohort totals*** | ***386.5*** | ***22*** | ***548*** | ***175.4*** | ***383*** | ***22*** | ***548*** | ***175.6*** | ***1*** | ***0*** | ***38*** | ***3.6*** | ***2*** | ***0*** | ***21*** | ***3.4*** |
| 266-MexTAg Het | 536.5 | 286 | 548 | 70.2 | 536 | 286 | 548 | 71.0 | 0 | 0 | 11 | 3.0 | 1.5 | 0.2 | 13.5 | 3.7 |
| 266-BEM_AG | 337 | 219 | 491 | 70.9 | 334 | 216 | 474 | 69.8 | 2 | 0 | 17 | 3.8 | 3 | 0.2 | 10 | 2.4 |
| 266-BOM_GB | 292 | 133 | 487 | 85.5 | 288.5 | 133 | 485 | 83.8 | 2 | 0 | 12 | 3.0 | 2 | 0.1 | 6 | 1.8 |
| 266-BOON_HF | 441 | 315 | 548 | 64.3 | 432.5 | 314 | 548 | 61.5 | 2 | 0 | 38 | 7.4 | 9 | 0.5 | 21 | 5.4 |
| 266-CAMERON_GA | 395 | 282 | 548 | 88.4 | 391 | 282 | 548 | 88.7 | 4 | 0 | 11 | 3.4 | 5.5 | 1 | 13 | 3.2 |
| 266-CC001/Unc | 475 | 332 | 548 | 71.4 | 470 | 329 | 548 | 72.3 | 1 | 0 | 7 | 2.4 | 2.75 | 0.2 | 10 | 2.9 |
| 266-CC017/Unc | 495 | 238 | 548 | 86.1 | 492 | 233 | 548 | 87.1 | 0 | 0 | 10 | 3.2 | 0.5 | 0 | 9 | 1.8 |
| 266-CC021/Unc | 548 | 359 | 548 | 59.0 | 548 | 359 | 548 | 59.6 | 0 | 0 | 12 | 2.8 | 4 | 0.4 | 14 | 3.3 |
| 266-CC035/Unc | 516 | 247 | 548 | 73.4 | 512 | 247 | 548 | 73.5 | 0 | 0 | 7 | 2.1 | 1.8 | 0.2 | 15 | 3.4 |
| 266-CC036/Unc | 548 | 412 | 548 | 47.0 | 548 | 412 | 548 | 47.7 | 0 | 0 | 13 | 2.9 | 2 | 0.1 | 9 | 2.8 |
| 266-CC040/TauUnc | 548 | 316 | 548 | 67.1 | 548 | 316 | 548 | 66.3 | 0 | 0 | 6 | 1.9 | 4 | 0.2 | 10 | 2.7 |
| 266-CC041/TauUnc | 548 | 420 | 548 | 23.1 | 548 | 417 | 548 | 23.6 | 0 | 0 | 3 | 0.7 | 2.2 | 0.1 | 6 | 1.4 |
| 266-CC044/Unc | 464.5 | 177 | 548 | 101.2 | 453 | 177 | 548 | 103.0 | 0 | 0 | 12 | 3.4 | 5.75 | 0.2 | 16 | 4.6 |
| 266-CC046/Unc | 538 | 353 | 548 | 62.1 | 525 | 353 | 548 | 62.5 | 0 | 0 | 13 | 2.8 | 2 | 0.5 | 10 | 2.4 |
| 266-CC053/Unc | 421 | 278 | 548 | 81.5 | 419 | 278 | 548 | 81.1 | 0 | 0 | 21 | 4.7 | 1 | 0.2 | 9 | 2.6 |
| 266-CC057/Unc | 427.5 | 182 | 548 | 109.7 | 418 | 182 | 548 | 109.2 | 2 | 0 | 13 | 3.7 | 2.5 | 0.1 | 9 | 2.4 |
| 266-CC058/Unc | 548 | 223 | 548 | 58.7 | 548 | 222 | 548 | 59.1 | 0 | 0 | 7 | 1.7 | 0.95 | 0.2 | 8.5 | 2.2 |
| 266-CC059/TauUnc | 546.5 | 249 | 548 | 92.1 | 546 | 243 | 548 | 93.7 | 0 | 0 | 27 | 5.1 | 5 | 0.3 | 10 | 2.7 |
| 266-CC060/Unc | 472 | 314 | 548 | 68.7 | 470 | 309 | 548 | 67.4 | 1 | 0 | 9 | 2.9 | 1.7 | 0.5 | 10 | 2.3 |
| 266-CC074/Unc | 485 | 309 | 548 | 63.3 | 481.5 | 309 | 548 | 64.8 | 1 | 0 | 17 | 4.1 | 6 | 0.5 | 14 | 4.9 |
| 266-CC081/Unc | 496 | 268 | 548 | 84.4 | 475 | 268 | 548 | 85.0 | 1 | 0 | 21 | 5.4 | 2.5 | 0.3 | 8 | 2.0 |
| 266-CIS_AD | 301 | 141 | 548 | 74.5 | 295 | 141 | 545 | 74.3 | 3 | 0 | 15 | 3.5 | 2.75 | 0.1 | 19 | 4.6 |
| 266-DAVIS_BA | 548 | 447 | 548 | 29.6 | 548 | 443 | 548 | 30.3 | 0 | 0 | 4 | 0.9 | 2.5 | 0.1 | 14 | 3.8 |
| 266-DONNELL_HA | 353 | 161 | 548 | 89.0 | 350 | 161 | 546 | 87.8 | 3 | 0 | 20 | 4.4 | 1.35 | 0.1 | 6 | 1.3 |
| 266-FEW_FD | 300 | 169 | 483 | 79.2 | 300.5 | 167 | 483 | 77.9 | 0 | 0 | 6 | 2.0 | 1.2 | 0.2 | 9 | 2.6 |
| 266FIM-DF | 293 | 207 | 524 | 70.0 | 285 | 203 | 521 | 69.9 | 3 | 0 | 14 | 4.5 | 1 | 0.2 | 11 | 2.9 |
| 266-FIV-AC | 393.5 | 263 | 544 | 61.1 | 391 | 260 | 538 | 60.4 | 1 | 0 | 7 | 1.9 | 1.95 | 0.2 | 11 | 2.9 |
| 266-FUF_HE | 268 | 171 | 359 | 49.8 | 261 | 164 | 356 | 49.7 | 3 | 0 | 8 | 2.6 | 2.25 | 0.1 | 8 | 2.5 |
| 266-GIG_EF | 375.5 | 226 | 548 | 92.2 | 371 | 225 | 548 | 91.8 | 3 | 0 | 9 | 2.9 | 4 | 0.5 | 8 | 2.2 |
| 266-GIT_GC | 312 | 165 | 434 | 67.1 | 308 | 163 | 420 | 66.5 | 3 | 0 | 14 | 3.2 | 2.5 | 0.1 | 18 | 5.4 |
| 266-HAX2_EF | 302 | 133 | 450 | 76.0 | 296.5 | 128 | 447 | 76.6 | 2 | 0 | 21 | 4.4 | 1.2 | 0.2 | 8.5 | 2.3 |
| 266-HAZ_FE | 329 | 162 | 548 | 112.8 | 327.5 | 158 | 546 | 112.0 | 2 | 0 | 7 | 2.4 | 5 | 0.7 | 14 | 3.9 |
| 266-HIP_GA | 209 | 22 | 373 | 79.0 | 207.5 | 22 | 370 | 78.3 | 3 | 0 | 32 | 8.0 | 5 | 0.5 | 15 | 3.9 |
| 266-JUD_EF | 314 | 217 | 418 | 57.0 | 314 | 215 | 418 | 57.8 | 3 | 0 | 24 | 5.3 | 0.875 | 0.1 | 13 | 3.2 |
| 266-JUNIOR_GB | 293 | 140 | 469 | 78.9 | 293 | 138 | 466 | 78.1 | 3 | 0 | 15 | 4.0 | 2.5 | 0.1 | 13 | 3.0 |
| 266-KAV_AF | 545 | 332 | 548 | 62.4 | 535 | 331 | 548 | 65.5 | 0 | 0 | 10 | 2.7 | 1 | 0.1 | 6 | 1.5 |
| 266-LAM_HD | 273 | 156 | 365 | 50.6 | 266 | 153 | 365 | 50.7 | 1 | 0 | 20 | 5.0 | 0.5 | 0.1 | 9 | 1.9 |
| 266-LAT_AC | 548 | 148 | 548 | 96.4 | 547 | 148 | 547 | 98.2 | 1 | 0 | 9 | 2.1 | 2 | 0.1 | 8 | 2.2 |
| 266-LAX_FC | 436 | 245 | 548 | 93.8 | 436 | 273 | 548 | 89.9 | 0 | 0 | 10 | 2.9 | 6 | 1.5 | 12 | 3.1 |
| 266-LEM_AF | 254 | 186 | 398 | 59.0 | 251.5 | 182 | 395 | 58.1 | 3 | 0 | 24 | 4.8 | 1 | 0.1 | 8 | 2.1 |
| 266-LEM2_AF | 318 | 215 | 548 | 85.1 | 306 | 215 | 548 | 82.8 | 3 | 0 | 21 | 5.3 | 1 | 0 | 15 | 4.5 |
| 266-LIL_AF | 548 | 380 | 548 | 54.6 | 548 | 380 | 548 | 54.9 | 0 | 0 | 5 | 1.2 | 2 | 0.1 | 11 | 2.8 |
| 266-LIV_DA | 266 | 156 | 380 | 53.4 | 265.5 | 156 | 370 | 53.5 | 2 | 0 | 21 | 5.5 | 3 | 0.1 | 10.5 | 2.9 |
| 266-LOD_AE | 301 | 108 | 506 | 96.3 | 298 | 108 | 503 | 95.5 | 3 | 0 | 17 | 3.6 | 4 | 0.1 | 12 | 3.4 |
| 266-LOT_FC | 411 | 271 | 548 | 82.5 | 408 | 270 | 548 | 84.7 | 1 | 0 | 17 | 4.2 | 1.05 | 0.1 | 12 | 3.0 |
| 266-LOX_GF | 548 | 408 | 548 | 46.6 | 548 | 405 | 548 | 46.6 | 0 | 0 | 11 | 2.6 | 4.5 | 0.1 | 15 | 3.6 |
| 266-LUF_AD | 236 | 131 | 412 | 81.1 | 225 | 131 | 407 | 79.5 | 2 | 0 | 17 | 5.2 | 1.5 | 0.1 | 8.2 | 2.7 |
| 266-LUS_AH | 405 | 269 | 548 | 80.9 | 408.5 | 269 | 548 | 80.3 | 3 | 0 | 14 | 3.8 | 0.65 | 0.1 | 10 | 2.5 |
| 266-LUV_DG | 477 | 184 | 548 | 93.1 | 472 | 184 | 548 | 94.0 | 1 | 0 | 10 | 3.0 | 1 | 0.1 | 10 | 2.5 |
| 266-MEE_AG | 548 | 226 | 548 | 80.9 | 548 | 226 | 548 | 85.0 | 0 | 0 | 5 | 1.0 | 1 | 0.2 | 8.5 | 2.1 |
| 266-NUK_AC | 548 | 331 | 548 | 61.9 | 541.5 | 331 | 548 | 62.7 | 0 | 0 | 12 | 3.4 | 3.5 | 0.8 | 11 | 2.3 |
| 266-PEF_EC | 410 | 173 | 548 | 98.8 | 407.5 | 169 | 548 | 99.9 | 2 | 0 | 12 | 2.4 | 0.95 | 0.1 | 4 | 1.2 |
| 266-PEF2_EC | 548 | 380 | 548 | 38.0 | 548 | 378 | 548 | 38.4 | 0 | 0 | 3 | 0.7 | 3 | 0.3 | 11 | 2.0 |
| 266-PIPING_BD | 315 | 123 | 509 | 107.1 | 313 | 123 | 509 | 106.5 | 2 | 0 | 9 | 2.5 | 3.75 | 0.5 | 14 | 4.8 |
| 266-POH_DC | 380.5 | 192 | 548 | 91.2 | 376 | 189 | 548 | 90.9 | 4 | 0 | 14 | 3.4 | 0.7 | 0.1 | 3 | 0.7 |
| 266-PUB_CD | 283 | 105 | 547 | 111.6 | 282 | 105 | 547 | 110.5 | 0 | 0 | 10 | 2.7 | 2 | 0.1 | 9 | 2.6 |
| 266-ROGAN_CF | 548 | 345 | 548 | 48.1 | 548 | 345 | 548 | 48.9 | 0 | 0 | 13 | 2.4 | 1.75 | 0 | 14 | 4.5 |
| 266-SAT_GA | 378 | 238 | 547 | 67.2 | 373 | 234 | 547 | 67.4 | 3 | 0 | 17 | 3.5 | 2.1 | 0 | 11.5 | 3.4 |
| 266-SHE_AH | 310 | 199 | 528 | 79.6 | 306 | 199 | 524 | 79.7 | 4 | 0 | 13 | 3.3 | 0.5 | 0 | 8.5 | 2.4 |
| 266-TAS_FE | 285.5 | 127 | 468 | 87.4 | 284 | 126 | 468 | 85.6 | 2 | 0 | 15 | 3.6 | 1.4 | 0 | 11 | 2.8 |
| 266-TOFU_FB | 206.5 | 96 | 548 | 141.0 | 204 | 96 | 546 | 140.8 | 1 | 0 | 8 | 2.1 | 3 | 0.2 | 13 | 3.5 |
| 266-TOP_DA | 422 | 152 | 548 | 102.8 | 421 | 152 | 548 | 102.7 | 0 | 0 | 10 | 2.5 | 1.5 | 0 | 8 | 2.6 |
| 266-VIT_ED | 267 | 125 | 547 | 108.0 | 263 | 125 | 547 | 107.2 | 1 | 0 | 12 | 3.5 | 2 | 0.1 | 11 | 3.9 |
| 266-VUX2_HF | 288.5 | 139 | 478 | 99.5 | 285.5 | 139 | 477 | 99.6 | 1 | 0 | 11 | 2.9 | 2.25 | 0.1 | 14 | 3.2 |
| 266-WAB2_DH | 239.5 | 115 | 433 | 90.9 | 239 | 115 | 428 | 89.3 | 1 | 0 | 11 | 3.0 | 3.25 | 0.5 | 11 | 3.8 |
| 266-WAD_HG | 522 | 313 | 548 | 85.4 | 494 | 312 | 548 | 84.4 | 0 | 0 | 9 | 2.6 | 2.5 | 0.2 | 19 | 4.7 |
| 266-WOB2_DH | 327.5 | 93 | 548 | 96.3 | 322.5 | 77 | 545 | 96.9 | 3 | 0 | 16 | 4.4 | 3 | 0 | 16.5 | 4.2 |
| 266-XAC2_HG | 146 | 104 | 318 | 63.9 | 144 | 104 | 366 | 77.6 | 1 | 0 | 10 | 2.9 | 1 | 0.1 | 13 | 2.7 |
| 266-XAK_AG | 344.5 | 240 | 548 | 73.6 | 341 | 239 | 548 | 73.0 | 2 | 0 | 14 | 3.5 | 3 | 0 | 13 | 4.0 |
| 266-YID_FH | 523 | 272 | 548 | 78.5 | 523 | 269 | 548 | 77.9 | 0 | 0 | 10 | 2.6 | 3 | 0.2 | 17 | 4.1 |
| 266-ZIE2_AH | 161 | 98 | 372 | 62.3 | 160.5 | 98 | 372 | 62.1 | 1 | 0 | 13 | 2.7 | 1 | 0 | 5.5 | 1.1 |
| 266-ZIF2_FC | 213 | 135 | 388 | 77.8 | 212 | 133 | 373 | 75.2 | 3 | 0 | 17 | 4.9 | 2 | 0 | 8 | 2.4 |
