# Supplementary material for: The MexTAg collaborative cross: host genetics affects asbestos related disease latency, but has little influence once tumours develop
Source: Front Toxicol. 2024 Apr 17;6:1373003. doi: 10.3389/ftox.2024.1373003 (PMC11061428; doi:10.3389/ftox.2024.1373003)
Supplement: Supplementary file 6 [file Table5.docx]

**Supplemental Table S5**: Genes located within ±10Mb of peak QTL associated with median ascites volume (ml).

| ***Chromosome*** | ***Gene ID*** | ***Gene Description*** |
| --- | --- | --- |
|  |  |  |
| **2** | *Kcna4* | potassium voltage-gated channel, shaker-related subfamily, member 4 |
| **3** | *Ptbp2* | polypyrimidine tract binding protein 2 |
|  | *Dpyd* | dihydropyrimidine dehydrogenase |
| **4** | *Dmap1* | DNA methyltransferase 1-associated protein 1 |
|  | *2610528J11Rik* | RIKEN cDNA 2610528J11 gene |
|  | *Rnf220* | ring finger protein 220 |
|  | *Dph2* | DPH2 homolog |
|  | *Ppcs* | phosphopantothenoylcysteine synthetase |
|  | *Med8* | mediator complex subunit 8 |
|  | *Tmem53* | transmembrane protein 53 |
|  | *Ebna1bp2* | EBNA1 binding protein 2 |
|  | *Svbp* | small vasohibin binding protein |
|  | *Ppih* | peptidyl prolyl isomerase H |
|  | *Hivep3* | human immunodeficiency virus type I enhancer binding protein 3 |
|  | *Slc6a9* | solute carrier family 6 (neurotransmitter transporter, glycine), member 9 |
|  | *Mpl* | myeloproliferative leukemia virus oncogene |
|  | *Rps8* | ribosomal protein S8 |
|  | *Slc2a1* | solute carrier family 2 (facilitated glucose transporter), member 1 |
|  | *Ermap* | erythroblast membrane-associated protein |
|  | *B4galt2* | UDP-Gal:betaGlcNAc beta 1,4- galactosyltransferase, polypeptide 2 |
|  | *Elovl1* | ELOVL fatty acid elongase 1 |
|  | *Cdc20* | cell division cycle 20 |
|  | *P3h1* | prolyl 3-hydroxylase 1 |
|  | *Tmem125* | transmembrane protein 125 |
|  | *Klf17* | Kruppel-like transcription factor 17 |
|  | *Foxj3* | forkhead box J3 |
|  | *Ccdc30* | coiled-coil domain containing 30 |
|  | *Kif2c* | kinesin family member 2C |
|  | *Tmem269* | transmembrane protein 269 |
|  | *Ybx1* | Y box protein 1 |
|  | *Zmynd12* | zinc finger, MYND domain containing 12 |
|  | *AU022252* | expressed sequence AU022252 |
|  | *Lao1* | L-amino acid oxidase 1 |
|  | *St3gal3* | ST3 beta-galactoside alpha-2,3-sialyltransferase 3 |
|  | *Artn* | artemin |
|  | *Or13p10* | olfactory receptor family 13 subfamily P member 10 |
|  | *Atp6v0b* | ATPase, H+ transporting, lysosomal V0 subunit B |
|  | *Eri3* | exoribonuclease 3 |
|  | *Tie1* | tyrosine kinase with immunoglobulin-like and EGF-like domains 1 |
|  | *Ptprf* | protein tyrosine phosphatase receptor type F |
|  | *Guca2a* | guanylate cyclase activator 2a (guanylin) |
|  | *Szt2* | SZT2 subunit of KICSTOR complex |
|  | *Or10ak7* | olfactory receptor family 10 subfamily AK member 7 |
|  | *Or10ak8* | olfactory receptor family 10 subfamily AK member 8 |
|  | *Or10ak16* | olfactory receptor family 10 subfamily AK member 16 |
|  | *Or10ak9* | olfactory receptor family 10 subfamily AK member 9 |
|  | *Or10ak11* | olfactory receptor family 10 subfamily AK member 11 |
|  | *Or10ak12* | olfactory receptor family 10 subfamily AK member 12 |
|  | *Or10ak13* | olfactory receptor family 10 subfamily AK member 13 |
|  | *Or10ak14* | olfactory receptor family 10 subfamily AK member 14 |
|  | *Or13p5* | olfactory receptor family 13 subfamily P member 5 |
|  | *Or13p8* | olfactory receptor family 13 subfamily P member 8 |
|  | *Or13p3* | olfactory receptor family 13 subfamily P member 3 |
|  | *Or13p4* | olfactory receptor family 13 subfamily P member 4 |
|  | *Cldn19* | claudin 19 |
|  | *Frg2f1* | FSHD region gene 2 family member 1 |
|  | *Rimkla* | ribosomal modification protein rimK-like family member A |
|  | *Zfp691* | zinc finger protein 691 |
|  | *Ipo13* | importin 13 |
|  | *Guca2b* | guanylate cyclase activator 2b (retina) |
|  | *Kdm4a* | lysine (K)-specific demethylase 4A |
|  | *Ccdc24* | coiled-coil domain containing 24 |
|  | *Cfap57* | cilia and flagella associated protein 57 |
|  | *Armh1* | armadillo-like helical domain containing 1 |
|  | *Klf18* | Kruppel-like transcription factor 18 |
|  | *Gm55960* | predicted gene, 55960 |
| **4** | *Taf12* | TATA-box binding protein associated factor 12 |
|  | *Rcc1* | regulator of chromosome condensation 1 |
|  | *Med18* | mediator complex subunit 18 |
|  | *Dhdds* | dehydrodolichyl diphosphate synthase |
|  | *Ubxn11* | UBX domain protein 11 |
|  | *Zfp593* | zinc finger protein 593 |
|  | *Dnajc8* | DnaJ heat shock protein family (Hsp40) member C8 |
|  | *Smpdl3b* | sphingomyelin phosphodiesterase, acid-like 3B |
|  | *Kdf1* | keratinocyte differentiation factor 1 |
|  | *Trnp1* | TMF1-regulated nuclear protein 1 |
|  | *Cd164l2* | CD164 sialomucin-like 2 |
|  | *Cep85* | centrosomal protein 85 |
|  | *Rps6ka1* | ribosomal protein S6 kinase polypeptide 1 |
|  | *Ptafr* | platelet-activating factor receptor |
|  | *Epb41* | erythrocyte membrane protein band 4.1 |
|  | *Fgr* | FGR proto-oncogene, Src family tyrosine kinase |
|  | *Hmgn2* | high mobility group nucleosomal binding domain 2 |
|  | *Oprd1* | opioid receptor, delta 1 |
|  | *Eya3* | EYA transcriptional coactivator and phosphatase 3 |
|  | *Tmem222* | transmembrane protein 222 |
|  | *Wasf2* | WASP family, member 2 |
|  | *Atp5if1* | ATP synthase inhibitory factor subunit 1 |
|  | *Crybg2* | crystallin beta-gamma domain containing 2 |
|  | *Rpa2* | replication protein A2 |
|  | *Cd52* | CD52 antigen |
|  | *Nr0b2* | nuclear receptor subfamily 0, group B, member 2 |
|  | *Mecr* | mitochondrial trans-2-enoyl-CoA reductase |
|  | *Map3k6* | mitogen-activated protein kinase kinase kinase 6 |
|  | *Sfn* | stratifin |
|  | *Srsf4* | serine and arginine-rich splicing factor 4 |
|  | *Lin28a* | lin-28 homolog A |
|  | *Sytl1* | synaptotagmin-like 1 |
|  | *Arid1a* | AT-rich interaction domain 1A |
|  | *Gmeb1* | glucocorticoid modulatory element binding protein 1 |
|  | *Themis2* | thymocyte selection associated family member 2 |
|  | *Rab42* | RAB42, member RAS oncogene family |
|  | *Pigv* | phosphatidylinositol glycan anchor biosynthesis, class V |
|  | *Gpatch3* | G patch domain containing 3 |
|  | *Ahdc1* | AT hook, DNA binding motif, containing 1 |
|  | *Ythdf2* | YTH N6-methyladenosine RNA binding protein 2 |
|  | *Trnau1ap* | tRNA selenocysteine 1 associated protein 1 |
|  | *Fam110d* | family with sequence similarity 110, member D |
|  | *Sh3bgrl3* | SH3 domain binding glutamic acid-rich protein-like 3 |
|  | *Tmem200b* | transmembrane protein 200B |
|  | *Zfp683* | zinc finger protein 683 |
|  | *Gm3627* | predicted gene 3627 |
|  | *Zdhhc18* | zinc finger, DHHC domain containing 18 |
|  | *Zfp593os* | Zfp593 opposite strand |
|  | *Zpld2* | zona pellucida like domain containing 2 |
|  | *Phactr4* | phosphatase and actin regulator 4 |
|  | *Gpn2* | GPN-loop GTPase 2 |
|  | *Ppp1r8* | protein phosphatase 1, regulatory subunit 8 |
|  | *Tent5b* | terminal nucleotidyltransferase 5B |
|  | *Ptpru* | protein tyrosine phosphatase receptor type U |
|  | *Nudc* | nudC nuclear distribution protein |
|  | *Gpr3* | G-protein coupled receptor 3 |
|  | *Slc9a1* | solute carrier family 9 (sodium/hydrogen exchanger), member 1 |
|  | *Gm30191* | predicted gene, 30191 |
|  | *Gm34296* | predicted gene, 34296 |
|  | *Catsper4* | cation channel, sperm associated 4 |
|  | *Stx12* | syntaxin 12 |
|  | *Sesn2* | sestrin 2 |
|  | *Cnksr1* | connector enhancer of kinase suppressor of Ras 1 |
|  | *Fam76a* | family with sequence similarity 76, member A |
|  | *Wdtc1* | WD and tetratricopeptide repeats 1 |
|  | *Xkr8* | X-linked Kx blood group related 8 |
|  | *Gm42323* | predicted gene, 42323 |
| **5** | *Speer4f1* | spermatogenesis associated glutamate (E)-rich protein 4F1 |
|  | *Cd36* | CD36 molecule |
|  | *Gnai1* | G protein subunit alpha i1 |
|  | *Hgf* | hepatocyte growth factor |
|  | *Cacna2d1* | calcium channel, voltage-dependent, alpha2/delta subunit 1 |
|  | *Sema3c* | sema domain, immunoglobulin domain (Ig), short basic domain, secreted, (semaphorin) 3C |
|  | *Gm10472* | predicted gene 10472 |
|  | *Gnat3* | G protein subunit alpha transducin 3 |
|  | *Cdhr17* | cadherin related family member 17 |
| **9** | *Abhd5* | abhydrolase domain containing 5 |
|  | *Snrk* | SNF related kinase |
|  | *Nktr* | natural killer tumor recognition sequence |
|  | *Vipr1* | vasoactive intestinal peptide receptor 1 |
|  | *Cyp8b1* | cytochrome P450, family 8, subfamily b, polypeptide 1 |
|  | *Ss18l2* | SS18, nBAF chromatin remodeling complex subunit like 2 |
|  | *Ackr2* | atypical chemokine receptor 2 |
|  | *Higd1a* | HIG1 domain family, member 1A |
|  | *Sec22c* | SEC22 homolog C, vesicle trafficking protein |
|  | *Klhl40* | kelch-like 40 |
|  | *Ccdc13* | coiled-coil domain containing 13 |
|  | *Hhatl* | hedgehog acyltransferase-like |
|  | *Xylb* | xylulokinase homolog (H. influenzae) |
|  | *Pomgnt2* | protein O-linked mannose beta 1,4-N-acetylglucosaminyltransferase 2 |
|  | *Gask1a* | golgi associated kinase 1A |
|  | *Zbtb47* | zinc finger and BTB domain containing 47 |
| **16** | *Atp5pf* | ATP synthase peripheral stalk subunit F6 |
|  | *App* | amyloid beta precursor protein |
|  | *Gabpa* | GA repeat binding protein, alpha |
|  | *Jam2* | junction adhesion molecule 2 |
|  | *Cyyr1* | cysteine and tyrosine-rich protein 1 |
|  | *Mrpl39* | mitochondrial ribosomal protein L39 |
|  | *A730009L09Rik* | RIKEN cDNA A730009L09 gene |
| **X** | *Rnf128* | ring finger protein 128 |
|  | *Tex13a* | testis expressed 13A |
|  | *Tbc1d8b* | TBC1 domain family, member 8B |
|  | *4933428M09Rik* | RIKEN cDNA 4933428M09 gene |
|  | *Nrk* | Nik related kinase |
|  | *Il1rapl2* | interleukin 1 receptor accessory protein-like 2 |
|  | *Trap1a* | tumor rejection antigen P1A |
|  | *Ripply1* | ripply transcriptional repressor 1 |
|  | *Morc4* | microrchidia 4 |
|  | *Radx* | RPA1 related single stranded DNA binding protein, X-linked |
|  | *Serpina7* | serine (or cysteine) peptidase inhibitor, clade A (alpha-1 antiproteinase, antitrypsin), member 7 |
|  | *Rbm41* | RNA binding motif protein 41 |
|  | *Pwwp3b* | PWWP domain containing 3B |
|  | *Cldn2* | claudin 2 |
