# Supplementary material for: The MexTAg collaborative cross: host genetics affects asbestos related disease latency, but has little influence once tumours develop
Source: Front Toxicol. 2024 Apr 17;6:1373003. doi: 10.3389/ftox.2024.1373003 (PMC11061428; doi:10.3389/ftox.2024.1373003)
Supplement: Supplementary file 7 [file Table3.docx]

**Supplemental Table S3**: Univariate analysis results for disease progression using general linear regression.

| **Feature** |  |  | **Mean Time (Days)** | **95 % CI** | | ***p*** |
| --- | --- | --- | --- | --- | --- | --- |
| Thickening |  | Absent |  |  |  | - |
|  |  | Present | 2.59 | 2.18 | 3.00 |  |
| Plaque |  | Absent | 1.37 | -0.53 | 3.27 | 0.195 |
|  |  | Present | 2.65 | 2.23 | 3.07 |  |
| Paucicellular |  | Absent | 2.77 | 2.27 | 3.27 | 0.203 |
|  |  | Present | 2.20 | 1.46 | 2.93 |  |
| Giant Cells |  | Absent | 2.77 | 2.32 | 3.22 | 0.055 |
|  |  | Present | 1.68 | 0.67 | 2.70 |  |
| Regular Nuclei |  | Absent | 0.89 | -1.06 | 2.84 | 0.079 |
|  |  | Present | 2.67 | 2.25 | 3.09 |  |
| Overt Tumour |  | Absent | 2.54 | 1.98 | 3.11 | 0.810 |
|  |  | Present | 2.65 | 2.04 | 3.25 |  |
| Hypercellularity |  | Absent | 0.83 | -2.55 | 4.21 | 0.304 |
|  |  | Present | 2.62 | 2.20 | 3.03 |  |
| Nuclear Atypia |  | Absent | 0.00 | -5.86 | 5.86 | 0.384 |
|  |  | Present | 2.60 | 2.19 | 3.02 |  |
| Mitotic Figures |  | Absent | 1.84 | 0.91 | 2.76 | 0.074 |
|  |  | Present | 2.78 | 2.32 | 3.24 |  |
| Invasion |  | Absent | 2.83 | 2.00 | 3.66 | 0.513 |
|  |  | Present | 2.51 | 2.04 | 2.99 |  |
