# Supplementary material for: The MexTAg collaborative cross: host genetics affects asbestos related disease latency, but has little influence once tumours develop
Source: Front Toxicol. 2024 Apr 17;6:1373003. doi: 10.3389/ftox.2024.1373003 (PMC11061428; doi:10.3389/ftox.2024.1373003)
Supplement: Supplementary file 8 [file Table6.docx]

**Supplemental Table S6: Univariate cox-regression analysis using the using the ‘coxph’ function from the ‘survival’ R package of 211 human mesothelioma patients in the Bueno dataset using age at the time of surgery as a surrogate for survival.** Only CCMT candidate genes with known human gene homologues were included in the analysis. Genes in **bold** (green highlight) had significant association with outcome. HR = Hazard ratio.

| **Covariates** | **beta** | **HR (95% CI for HR)** | **wald.test** | **p.value** |
| --- | --- | --- | --- | --- |
| Sex | 0.021 | 1 (0.66-1.6) | 0.01 | 0.93 |
| *CFTR* | 0.083 | 1.1 (0.8-1.5) | 0.27 | 0.6 |
| *WNT16* | 0.054 | 1.1 (0.77-1.4) | 0.12 | 0.73 |
| *ST7* | 0.17 | 1.2 (0.87-1.6) | 1.1 | 0.29 |
| *SLC25A5* | 0.092 | 1.1 (0.8-1.5) | 0.34 | 0.56 |
| *LAMP2* | 0.29 | 1.3 (0.97-1.8) | 3.2 | 0.072 |
| *STEEP1* | 0.049 | 1.1 (0.77-1.4) | 0.1 | 0.76 |
| *PUM2* | 0.13 | 1.1 (0.83-1.6) | 0.63 | 0.43 |
| *LAPTM4A* | 0.19 | 1.2 (0.88-1.6) | 1.4 | 0.24 |
| *ING3* | 0.026 | 1 (0.75-1.4) | 0.03 | 0.87 |
| *CTTNBP2* | 0.089 | 1.1 (0.8-1.5) | 0.32 | 0.57 |
| *SLC25A43* | -0.0073 | 0.99 (0.73-1.4) | 0 | 0.96 |
| *UBE2A* | 0.26 | 1.3 (0.95-1.8) | 2.7 | 0.099 |
| *DDX1* | -0.12 | 0.89 (0.65-1.2) | 0.55 | 0.46 |
| *APOB* | 0.024 | 1 (0.75-1.4) | 0.02 | 0.88 |
| *PGRMC1* | 0.01 | 1 (0.74-1.4) | 0 | 0.95 |
| *NKAP* | 0.042 | 1 (0.76-1.4) | 0.07 | 0.79 |
| *RHOXF1* | -0.013 | 0.99 (0.72-1.3) | 0.01 | 0.93 |
| *ATP1B4* | 0.0033 | 1 (0.73-1.4) | 0 | 0.98 |
| *CAV2* | -0.013 | 0.99 (0.72-1.3) | 0.01 | 0.94 |
| *CAV1* | 0.08 | 1.1 (0.79-1.5) | 0.25 | 0.61 |
| *MET* | 0.096 | 1.1 (0.81-1.5) | 0.37 | 0.54 |
| *WNT2* | 0.12 | 1.1 (0.82-1.5) | 0.54 | 0.46 |
| *TSPAN12* | 0.11 | 1.1 (0.82-1.5) | 0.5 | 0.48 |
| ***CPED1*** | **0.38** | **1.5 (1.1-2)** | **5.8** | **0.016** |
| *PTPRZ1* | 0.1 | 1.1 (0.81-1.5) | 0.41 | 0.52 |
| *SDC1* | -0.14 | 0.87 (0.64-1.2) | 0.73 | 0.39 |
| *HS1BP3* | 0.26 | 1.3 (0.96-1.8) | 2.8 | 0.094 |
| *LDAH* | 0.18 | 1.2 (0.87-1.6) | 1.2 | 0.27 |
| *WDR35* | 0.1 | 1.1 (0.81-1.5) | 0.42 | 0.51 |
| *UPF3B* | 0.0084 | 1 (0.74-1.4) | 0 | 0.96 |
| *RNF113A* | 0.012 | 1 (0.74-1.4) | 0.01 | 0.94 |
| *SEPTIN6* | 0.12 | 1.1 (0.83-1.5) | 0.6 | 0.44 |
| *TMEM255A* | -0.12 | 0.89 (0.65-1.2) | 0.57 | 0.45 |
| *NDUFA1* | -0.28 | 0.76 (0.56-1) | 3.1 | 0.08 |
| *LSM8* | 0.18 | 1.2 (0.88-1.6) | 1.3 | 0.26 |
| *IL13RA1* | 0.15 | 1.2 (0.85-1.6) | 0.88 | 0.35 |
| *MATN3* | 0.023 | 1 (0.75-1.4) | 0.02 | 0.89 |
| *TES* | 0.18 | 1.2 (0.87-1.6) | 1.2 | 0.27 |
| *SYCP3* | -0.05 | 0.95 (0.69-1.3) | 0.09 | 0.76 |
| ***OSR1*** | **0.35** | **1.4 (1-1.9)** | **5** | **0.026** |
| *GDF7* | -0.17 | 0.85 (0.62-1.2) | 1.1 | 0.29 |
| *RHOB* | -0.11 | 0.89 (0.65-1.2) | 0.52 | 0.47 |
| *DOCK11* | 0.24 | 1.3 (0.93-1.7) | 2.3 | 0.13 |
| *NBAS* | 0.14 | 1.2 (0.84-1.6) | 0.81 | 0.37 |
| *ASZ1* | 0.092 | 1.1 (0.8-1.5) | 0.33 | 0.57 |
| *CUL4B* | 0.095 | 1.1 (0.8-1.5) | 0.35 | 0.56 |
| *SMC6* | 0.0097 | 1 (0.74-1.4) | 0 | 0.95 |
| *VSNL1* | 0.086 | 1.1 (0.8-1.5) | 0.3 | 0.59 |
| *ZCCHC18* | 0.15 | 1.2 (0.85-1.6) | 0.89 | 0.35 |
| *KCNS3* | 0.01 | 1 (0.74-1.4) | 0 | 0.95 |
| *C1GALT1C1* | 0.028 | 1 (0.75-1.4) | 0.03 | 0.86 |
| *ZCCHC12* | -0.22 | 0.8 (0.59-1.1) | 1.9 | 0.16 |
| *LONRF3* | 0.16 | 1.2 (0.86-1.6) | 1.1 | 0.3 |
| *ZBTB33* | -0.057 | 0.94 (0.69-1.3) | 0.12 | 0.73 |
| *GEN1* | -0.2 | 0.82 (0.6-1.1) | 1.5 | 0.22 |
| *TTC32* | -0.089 | 0.91 (0.67-1.2) | 0.31 | 0.57 |
| *KCND2* | -0.012 | 0.99 (0.72-1.3) | 0.01 | 0.94 |
| *NT5C1B* | 0.085 | 1.1 (0.8-1.5) | 0.29 | 0.59 |
| *SHOX* | 0.15 | 1.2 (0.85-1.6) | 0.85 | 0.36 |
| *NKRF* | 0.11 | 1.1 (0.81-1.5) | 0.46 | 0.5 |
| *SOWAHD* | 0.29 | 1.3 (0.98-1.8) | 3.3 | 0.069 |
| *FAM3C* | 0.15 | 1.2 (0.86-1.6) | 0.94 | 0.33 |
| *CYRIA* | 0.22 | 1.2 (0.92-1.7) | 2 | 0.16 |
| *CAPZA2* | 0.19 | 1.2 (0.88-1.7) | 1.4 | 0.23 |
| *RPL39* | -0.26 | 0.77 (0.57-1.1) | 2.7 | 0.1 |
| *RAD51AP2* | 0.041 | 1 (0.76-1.4) | 0.07 | 0.8 |
| *MCTS1* | -0.094 | 0.91 (0.67-1.2) | 0.35 | 0.55 |
| *RDH14* | -0.079 | 0.92 (0.68-1.3) | 0.25 | 0.62 |
| *KIAA1210* | -0.026 | 0.97 (0.71-1.3) | 0.03 | 0.87 |
